# Poverty and Social Disadvantage in Women and Men and Fertility Outcomes

**DOI:** 10.1001/jamanetworkopen.2025.32741

**Published:** 2025-09-19

**Authors:** Aline J. Boxem, Annemarie G. M. G. J. Mulders, Iris van Rossum, Eline L. Bekkers, Romy Gaillard, Vincent W. V. Jaddoe

**Affiliations:** 1 The Generation R Study Group, Erasmus MC, University Medical Centre, Rotterdam, the Netherlands; 2 Department of Pediatrics, Erasmus MC, University Medical Centre, Rotterdam, the Netherlands; 3 Department of Obstetrics and Gynecology, Erasmus MC, University Medical Centre, Rotterdam, the Netherlands

## Abstract

**Importance:**

Poverty and social disadvantage are associated with increased risks of pregnancy complications. It is not known whether poverty and social disadvantage are associated with subfertility and miscarriage risks.

**Objective:**

To assess whether poverty and markers of social disadvantage, including low educational level and household income among both women and men, are associated with subfertility and miscarriage risks.

**Design, Setting, and Participants:**

This population-based prospective cohort study, which was conducted in an urban setting in Rotterdam, the Netherlands, between August 9, 2017, and July 1, 2021, included women and their partners from the preconception period onward with follow-up until birth. The date of analysis was July 8, 2025.

**Exposures:**

Poverty, defined as a household income of less than €3000 per month and experiencing financial difficulties; educational level; and household income.

**Main Outcomes and Measures:**

The main outcomes were fecundability, defined as the per-month probability of conceiving; subfertility, defined as a time to pregnancy or the duration of actively pursuing pregnancy of more than 12 months or use of assisted reproductive technology; and miscarriage, defined as pregnancy loss before 22 weeks’ gestation. Data were analyzed using Cox proportional hazards regression and modified Poisson regression models.

**Results:**

Among 3604 women (median age, 31.2 [IQR, 28.5-34.3] years) and 2557 male partners (median age, 33.2 [IQR, 30.0-36.6] years), the time-to-pregnancy study population consisted of 2851 episodes among women (median age, 31.5 [IQR, 29.1-34.4] years) and 2830 episodes among men (median age, 33.3 [IQR, 30.4-36.7] years). The miscarriage study population consisted of 2515 episodes among women (median age, 31.3 [IQR, 28.8-34.1] years) and 2498 episodes among men (median age, 33.3 [IQR, 30.1-36.5] years). Among women, the median time to pregnancy was 3.5 months (95% range [2.5%-97.5%], 0-67.8 months); in total, 974 episodes (34.6%) were subfertile, and 297 pregnancy episodes (11.8%) led to a miscarriage. Subanalyses were based on 2103 to 2805 episodes among women. Poverty was associated with lower fecundability (confounder model fecundability ratio [FR], 0.61 [95% CI, 0.51-0.72]). Also, as compared with high educational level, low educational level among women (FR, 0.61 [95% CI, 0.50-0.74]) and men (FR, 0.72 [95% CI, 0.62-0.85]) was associated with lower fecundability. As compared with a household income of €6000 or more per month, a household income of less than €3000 per month was associated with lower fecundability (FR, 0.59 [95% CI, 0.51-0.68]). The effect estimates of subfertility were in line with those for fecundability; for example, poverty was associated with increased subfertility risk (32.5% vs 50.3%; relative risk, 1.37 [95% CI, 1.16-1.62]). These associations were only partly explained by demographic and lifestyle factors. Poverty and markers of social disadvantage were not associated with miscarriage risk.

**Conclusions and Relevance:**

The findings of this cohort study suggest that poverty and social disadvantage, characterized by low educational level and household income among both women and men, were associated with lower fecundability and increased risks of subfertility but not with miscarriage risk. Further studies are needed to identify the underlying and explanatory mechanisms associated with fertility outcomes and the potential for novel public health strategies for couples desiring pregnancy.

## Key Points

**Question:**

Are poverty and social disadvantage, defined by low educational level and household income, associated with lower fecundability and higher risks of subfertility and miscarriage?

**Findings:**

In this cohort study of 3604 women and their partners that was conducted in an urban high-income setting, poverty and social disadvantage were associated with lower fecundability and higher risks of subfertility but not with higher risks of miscarriage.

**Meaning:**

These findings suggest that poverty and social disadvantage are associated with fertility outcomes; further studies are needed to identify the underlying and explanatory mechanisms associated with fertility outcomes and the potential for novel public health strategies for couples desiring pregnancy.

## Teaser

This cohort study assesses whether poverty and markers of social disadvantage, including low educational level and household income among women and men, are associated with higher risks of subfertility and miscarriage and lower fecundability.

## Introduction

Poverty has a great impact on long-term health outcomes from the early stages of life onward and is a risk factor for pregnancy complications.^1,2,3,4,5,6,7,8^ Previous studies have shown that in high-income countries, poverty as well as lower educational level and income among women are associated with increased risks of pregnancy complications, such as preeclampsia, gestational diabetes, preterm birth, and neonatal death.^9,10^ Pregnancy and birth complications often originate in suboptimal embryonic and placental development in early pregnancy.^11,12,13,14^ Results from studies in Denmark^15^ and the US^17^ suggest that couples’ lower educational level reduces fecundability, possibly explained by practical and financial barriers to receive reproductive health counseling.^16^ These studies did not assess associations with other fertility and early-pregnancy outcomes. The association of educational level with fecundability might be U-shaped because of the presence of different lifestyle and age-related risk factors at both ends of the distribution.^18^ Thus far, the reported associations of low household income with fertility are inconsistent.^19,20^ A report from the Global Burden of Disease data from 204 countries reported that women’s highest educational level was associated with lower miscarriage risk.^21^ However, this was a cross-sectional register-based study with lack of data standardization and quality control uncertainties. Knowledge about the potential reproductive consequences of poverty and social disadvantage among women and men may contribute to targeted preventive strategies for adverse reproductive outcomes and pregnancy complications.

We hypothesized that poverty and social disadvantage among both women and men would be associated with subfertility and miscarriage risks. In this population-based prospective cohort study in a high-income setting from preconception onward among women and their male partners, we assessed the associations of poverty and social disadvantage, including low educational level and household income, with fecundability and subfertility and miscarriage risks, and whether any association was explained by demographic and lifestyle factors.

## Methods

### Study Design

This cohort study was embedded in the Generation R Next study, a population-based prospective cohort study from the preconception period onward in Rotterdam, the Netherlands.^22^ The study method and measurements timeline are described in eFigure 1 in Supplement 1. Women and their partners in the general population were eligible if they were 18 years or older, living in Rotterdam, and were actively trying to conceive or were pregnant. They were recruited between August 9, 2017, and July 1, 2021, through various approaches: information folders sent to all inhabitants living in the city of Rotterdam; information about the study through their general practitioner, pharmacy, obstetric caregiver, or acquaintances; and social media, posters, and news outlets. Inclusion was intended for preconception or early pregnancy but was allowed until delivery. In total, 33.2% of episodes were included during preconception, and 52.8% of episodes were included during the first trimester of pregnancy. Study approval was obtained by the Medical Ethical Committee of the Erasmus MC, and written informed consent was obtained from all participants. This study followed the Strengthening the Reporting of Observational Studies in Epidemiology (STROBE) reporting guideline.

During the study, couples could participate multiple times with different preconception and pregnancy episodes. In total, 71.3% of the women’s partners participated. In this study, due to related data of women participating multiple times, only the first episode was included, and female partners were excluded. After including only first episodes and excluding episodes with missing outcome data, analyses were based on 2662 to 2805 episodes for time-to-pregnancy and subfertility analyses and 2103 to 2285 episodes for miscarriage analyses (eFigures 2 and 3 in Supplement 1).

### Poverty and Social Disadvantage

Information on educational level, household income, and experiencing financial difficulties was obtained through self-reported questionnaires at enrollment. To assess experiencing financial difficulties, participants reported to what extent their household income covered their usual monthly expenses. Poverty was defined as yes (<€3000 per month combined with experiencing financial difficulties) or no (<€3000 per month combined with not experiencing financial difficulties or ≥€3000 per month, combined with experiencing or not experiencing financial difficulties), based on Dutch poverty measures.^23,24^ Information on the male partner’s educational level was self-reported or, if he did not participate, reported by the woman. Educational level was categorized using the International Standard Classification of Education levels: low (0-2), middle (3-5), and high (6-8) (eMethods in Supplement 1).^25,26^ Couples’ educational level was categorized as both without high, only women high, only men high, or both high. Low and middle educational level were merged due to small sample sizes. Household income was categorized as less than €3000 (poverty level), €3000 to €5999, and €6000 or more per month, reflecting the Dutch modal household income for 2 adults.^27^

### Time to Pregnancy and Miscarriage

As previously noted, time to pregnancy and mode of conception were assessed through questionnaires in preconception and early pregnancy.^22^ Participants reported the date at which they started trying to conceive and stopped using contraceptives (start date pursuing pregnancy) and the assisted reproductive technology date, including intrauterine insemination, ovulation induction, in vitro fertilization, and intracytoplasmic sperm injection. Participants included during preconception informed the study when obtaining a self-performed positive pregnancy test and provided the date of the woman’s last menstrual period (LMP). Participants included during the first trimester reported the LMP date at enrollment and in questionnaires, and this was reconfirmed during first-trimester visits to the obstetric caregiver using embryo size, which translated to estimates of LMP, term date, and pregnancy duration. In women who conceived, time to pregnancy was calculated from the start date to the LMP date. In women who did not conceive, the duration of pursuing pregnancy was calculated from the start date to the last day of study participation (ie, the date when voluntarily withdrawing) or the study end date. Inclusion of participants ended on December 31, 2020, and participants were followed up until July 1, 2021 (study end date). Ongoing pregnancies at that point were followed up until birth. Time to pregnancy was assessed continuously and categorized. Continuous time to pregnancy was used to assess fecundability, which is the per-month probability of conceiving, defined as 4 weeks (28 days), to represent a regular menstrual cycle length. Time to pregnancy was categorized as fertile (0-52 weeks [12 months]) or infertile (time to pregnancy or, in cases of not conceiving, duration of pursuing pregnancy of >52 weeks [>12 months] or use of assisted reproductive technology), according to the clinical definition.^28,29,30,31^ Spontaneous miscarriage was defined as a pregnancy loss before 22 weeks’ gestation.^32^ Miscarriage date and gestational age of miscarriage were obtained from the obstetric caregiver using LMP dates and due date based on ultrasonography, the date of intrauterine insemination, or embryo implant day minus 14 days in cases of in vitro fertilization or intracytoplasmic sperm injection.

### Potential Explanatory and Demographic Factors

Information on age, parity, and history of previous miscarriage was assessed through questionnaires during preconception and/or early pregnancy. Information on migration background was obtained through questionnaires completed in writing by the participant at enrollment and was included in the analysis since it may be associated with the exposure and outcomes.^33,34^ Migration background is known to be associated with differences in markers of social disadvantage.^35,36^ Migration background was based on questions regarding participants’ and their parents’ birth country, classified according to Statistics Netherlands.^37,38^ If either parent was born outside of the Netherlands, participants were classified as having a non-Dutch migration background. If both parents were born outside of the Netherlands, the mothers’ birth country defined the participants’ migration background. Cohabitation status was defined as cohabiting or not cohabiting.

### Potential Lifestyle Factors

Information on folic acid supplementation, smoking, and alcohol consumption was assessed through questionnaires in preconception or early-pregnancy periods. Information on body mass index was obtained from height and weight measurements at enrollment or, if missing, subsequent preconceptional and/or first-trimester visits.

### Statistical Analysis

The date of analysis was July 8, 2025. First, we performed a nonresponse analysis comparing characteristics of participants with and without time-to-pregnancy or miscarriage data using *t* tests, Mann-Whitney tests, χ^2^ tests, or Fisher exact tests. Second, we examined associations of poverty, educational level, and household income with fecundability and miscarriage using Cox proportional hazards regression models (package survival in R, version 3.6-4 [R Project for Statistical Computing]). The fecundability outcome was conception or no conception, and the time variable was based on time to pregnancy among women with conception or duration of actively pursuing pregnancy among women without conception, excluding women undergoing assisted conception due to unknown time to pregnancy. For miscarriage, the outcome was miscarriage or no miscarriage, and the time variable was based on gestational age in weeks. We checked model assumptions using Schoenfeld residual plots and Martingale and deviance residuals. The resulting hazard ratio (HR) represents the fecundability ratio, which compares the per-cycle probability of conception between exposure categories; a fecundability ratio (FR) less than 1 indicates reduced fecundability compared with the reference group (eMethods in Supplement 1). Fecundability and miscarriage risks were graphically assessed using Kaplan-Meier curves, and effect estimates with 95% CIs were presented within the figures. Third, we examined associations of poverty, educational level, and household income with subfertility and miscarriage risks using modified Poisson regression models with robust SEs, as recommended by Zou.^39^ Model assumptions were checked, including linearity, influential points, and estimated probabilities. The linearity assumption for age of women was violated in the subfertility analyses, and therefore, all models included a quadratic age term. All models were first adjusted for age and parity in the subfertility models and for age, parity, and history of miscarriage in the miscarriage models. Next, if any association was present, we added potential explanatory factors. We performed 3 sensitivity analyses. First, to account for the right-skewed time-to-pregnancy distribution, the Cox proportional hazards regression analysis was repeated, excluding the top 5% of the time-to-pregnancy distribution. Second, to assess whether any association was influenced by the use of assisted conception, we excluded participants who underwent assisted reproductive technology. Third, to assess whether any association was influenced by migration background, we repeated the analyses among only Dutch participants. Missing values of potential explanatory variables were imputed using multiple imputation by chained equations to reduce potential bias (package mice in R, version 3.17.0). Due to the likely not-missing-at-random nature of the exposure, we did not impute the exposures. Pooled results were reported. Analyses were performed using R, version 4.4.1. Two-sided *P* < .05 was considered significant.

## Results

### Population Characteristics

The total study population consisted of 3604 unique women (median age, 31.2 [IQR, 28.5-34.3 years]) and 2557 male partners (median age, 33.2 [IQR, 30.0-36.6] years), with a total of 4036 participant episodes leading to 3577 pregnancy episodes. Table 1 shows that the time-to-pregnancy study population consisted of 2851 episodes among women (median age, 31.5 [IQR, 29.1-34.4] years) and 2830 episodes among men (median age, 33.3 [IQR, 30.4-36.7] years). The miscarriage study population consisted of 2515 episodes among women (median age, 31.3 [IQR, 28.8-34.1] years) and 2498 episodes among men (median age, 33.3 [IQR, 30.1-36.5] years). Among women, the median time to pregnancy was 3.5 months (95% range [2.5%-97.5%], 0-67.8 months). In total, 974 episodes (34.6%) were subfertile, and 297 pregnancy episodes (11.8%) led to a miscarriage. Detailed information of characteristics per subanalysis are provided in eTable 1 in Supplement 1. Characteristics across exposure levels are provided in eTables 2-5 in Supplement 1, and characteristics of participants with and without the observed outcomes with a loss to follow-up of 5.1% (163 of 3181) are provided in eTable 6 in Supplement 1. As compared with participants who were not included in the study, those included were older, had a higher household income per month, were more frequently cohabiting, and started folic acid supplementation more frequently before pregnancy. The percentage of women with 1 or more missing covariates was 14.9%, and the percentage of men with 1 or more missing covariates was 32.2%; missing data ranged from 0% to 25.8% (Table 1).

**Table 1.** Participant Characteristics^a^

Characteristic	Episodes			
	Time to pregnancy		Miscarriage	
	Women (n = 2851)^b^	Men (n = 2830)^c^	Women (n = 2515)^d^	Men (n = 2498)^c^
Age at enrollment				
Median (IQR), y	31.5 (29.1-34.4)	33.3 (30.4-36.7)	31.3 (28.8-34.1)	33.3 (30.1-36.5)
Missing	0	641 (22.7)	0	407 (16.3)
Migration background				
Dutch	1728 (61.2)	1626 (61.9)	1433 (62.2)	1353 (62.2)
European^e^	290 (10.3)	222 (8.5)	226 (9.8)	172 (7.9)
Non-European^f^	805 (28.5)	779 (29.7)	645 (28.0)	649 (29.9)
Missing	28 (1.0)	203 (7.2)	211 (8.4)	324 (13.0)
Poverty^g^				
Yes	169 (6.3)	168 (6.4)	127 (6.0)	126 (6.0)
No	2493 (93.7)	2476 (93.6)	1976 (94.0)	1962 (94.0)
Missing	189 (6.6)	186 (6.6)	412 (16.4)	410 (16.4)
Educational level^h^				
Low	147 (5.2)	238 (8.9)	120 (5.3)	196 (9.0)
Middle	669 (23.9)	804 (30.1)	549 (24.0)	637 (29.2)
High	1989 (70.9)	1625 (60.9)	1616 (70.7)	1350 (61.8)
Missing	46 (1.6)	163 (5.8)	230 (9.1)	315 (12.6)
Household income per mo, €^i^				
<3000	664 (24.1)	658 (24.1)	527 (24.0)	522 (23.9)
3000-5999	1697 (61.7)	1683 (61.6)	1351 (61.4)	1340 (61.4)
≥6000	391 (14.2)	390 (14.3)	322 (14.6)	321 (14.7)
Missing	99 (3.5)	99 (3.5)	315 (12.5)	315 (12.6)
Cohabitation status^j^				
Not cohabiting	331 (12.3)	330 (12.3)	244 (11.5)	243 (11.5)
Cohabiting	2362 (87.7)	2345 (87.7)	1884 (88.5)	1870 (88.5)
Missing	158 (5.5)	155 (5.5)	387 (15.4)	385 (15.4)
BMI				
Median (IQR)	23.4 (21.2-26.5)	24.9 (23.0-27.4)	23.5 (21.3-26.7)	24.9 (23.0-27.5)
Missing	41 (1.4)	731 (25.8)	33 (1.3)	526 (21.1)
Smoking				
No	1541 (55.7)	1301 (52.4)	1226 (55.8)	1024 (50.2)
No (quit smoking before pregnancy)	856 (30.9)	565 (22.8)	650 (29.6)	496 (24.3)
Yes (smoked during pregnancy)	372 (13.4)	615 (24.8)	323 (14.7)	521 (25.5)
Missing	82 (2.9)	349 (12.3)	316 (12.6)	457 (18.3)
Alcohol consumption				
No consumption <3 mo before pregnancy	596 (21.5)	348 (13.3)	486 (21.9)	267 (12.9)
Yes (consumption <3 mo before pregnancy)	1776 (64.1)	2269 (86.7)	1354 (61.1)	1810 (87.1)
Yes (consumption during pregnancy)	397 (14.3)	NA	376 (17.0)	NA
Missing	82 (2.9)	213 (7.5)	299 (11.9)	421 (16.9)
Folic acid supplementation				
Never	26 (1.0)	26 (1.0)	20 (0.9)	20 (0.9)
Started prior to pregnancy	1770 (67.7)	1754 (67.6)	1420 (66.9)	1406 (66.8)
Started in pregnancy	817 (31.3)	816 (31.4)	681 (32.1)	680 (32.3)
Missing	238 (8.3)	234 (8.3)	394 (15.7)	392 (15.7)
Parity				
Nulliparous	1871 (68.0)	1856 (67.9)	1486 (67.3)	1474 (67.2)
Multiparous	880 (32.0)	877 (32.1)	722 (32.7)	719 (32.8)
Missing	100 (3.5)	97 (3.4)	307 (12.2)	305 (12.2)
Miscarriage in previous pregnancy				
No	2231 (80.8)	2212 (80.6)	1776 (81.5)	1760 (81.3)
Yes	531 (19.2)	531 (19.4)	404 (18.5)	404 (18.7)
Missing	89 (3.1)	87 (3.1)	335 (13.3)	334 (13.4)
Time to pregnancy, mo^k^				
Median (95% range [2.5%-97.5%])	3.5 (0-67.8)	3.5 (0-67.3)	3.3 (0-61.5)	3.3 (0-61.0)
0-12	1842 (64.6)	1838 (64.9)	1543 (72.2)	1539 (72.6)
>12	477 (16.7)	474 (16.7)	362 (16.9)	359 (16.9)
ART leading to pregnancy	283 (9.9)	270 (9.5)	232 (10.9)	222 (10.5)
Not pregnant	249 (8.7)	248 (8.8)	NA	NA
Overall subfertility^l^	974 (34.6)	957 (34.2)	594 (27.8)	581 (27.4)
Missing	0	0	378 (15.0)	378 (15.1)
Occurrence of miscarriage				
No miscarriage	2399 (92.2)	2385 (92.4)	2218 (88.2)	2207 (88.4)
Miscarriage	203 (7.8)	197 (7.6)	297 (11.8)	291 (11.6)
Missing	249 (8.7)	248 (8.8)	0	0
Timing of miscarriage, wk				
Median (IQR)	8.1 (7.0-9.6)	8.1 (7.0-9.5)	8.3 (7.1-9.4)	8.3 (7.1-9.4)
First trimester	187 (93.0)	182 (93.3)	272 (93.2)	267 (93.4)
Second trimester	14 (7.0)	13 (6.7)	20 (6.8)	19 (6.6)
Missing	2 (1.0)	2 (1.0)	5 (1.7)	5 (1.7)

Abbreviations: ART, assisted reproductive technology; BMI, body mass index (calculated as weight in kilograms divided by height in meters squared); NA, not applicable.

^a^ Data are presented as the No. (%) of participants unless indicated otherwise. The total study population consisted of 3604 unique women from Rotterdam, the Netherlands, with a total of 4036 participant episodes leading to 3577 pregnancy episodes. Women were included in preconception and pregnancy between August 9, 2017, and July 1, 2021.

^b^ The study population of time to pregnancy consisted of 2851 unique women from Rotterdam, the Netherlands.

^c^ The study population of time to pregnancy and miscarriage consisted of 2830 and 2498 unique men from Rotterdam, the Netherlands, respectively. Poverty, household income, cohabitation status, folic acid supplementation, parity, miscarriage in previous pregnancy, time to pregnancy in months, occurrence of miscarriage, timing of miscarriage in weeks were derived from their partner.

^d^ The study population of miscarriage consisted of 2515 unique women from Rotterdam, the Netherlands.

^e^ Includes European, German, Polish, or Yugoslav.

^f^ Includes African; American, non-Western and Western; Asian, non-Western and Western; Cape Verdean; Chinese; Dutch Antilles; Indonesian; Moroccan; Oceanian; Surinamese; or Turkish.

^g^ Yes: experiencing financial difficulties combined with a household income of less than €3000 per month. No: experiencing financial difficulties combined with a household income of €3000 to €5999 per month or €6000 or more per month or not experiencing financial difficulties combined with household income of less than €3000 per month; €3000 to €5999 per month and equal to or more than €6000 per month.

^h^ Low: no primary education finished, primary education finished, and secondary education phase 1 finished. Middle: secondary education phase 2 finished. High: higher education phase 1 finished and higher education phase 2 finished.

^i^ Less than €3000 per month: €999 or less per month, €1000 to €1999 per month, and €2000 to €2999 per month. €3000 to €5999 per month: €3000 to €3999 per month, €4000 to €4999 per month, and €5000 to €5999 per month.

^j^ Not cohabiting: no partner and/or not cohabiting and married, living separately. Cohabiting: cohabiting but not married; married and cohabiting with spouse; and registered partnership and cohabiting.

^k^ Derived from pregnancy episodes with a natural conception.

^l^ Episodes with ART leading to pregnancy; episodes without pregnancy and use of ART (8 of 249 episodes); and episodes without pregnancy, without use of ART, and a duration of actively pursuing pregnancy of more than 12 months (206 of 249 episodes) were added to the subfertile group (time to pregnancy >12 months and use of ART) in the analysis.

### Fecundability and Subfertility

**Figure 1.**
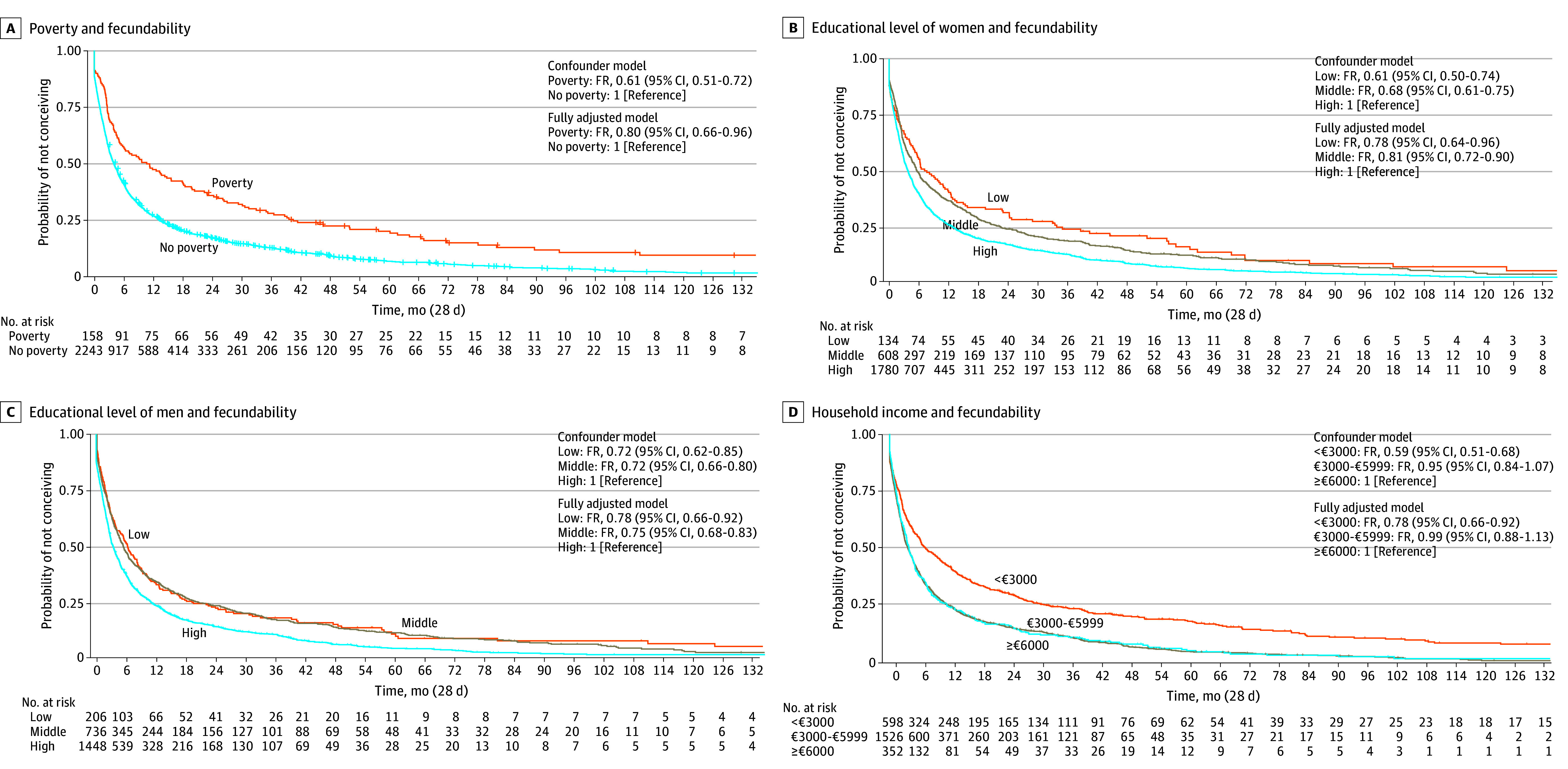
Associations of Poverty, Educational Level of Women and Men, and Household Income With Fecundability Fecundability ratios (FRs) with 95% CIs of poverty (A), of educational level (B and C), and household income (D) categories associated with fecundability. FRs were derived from the HRs of the Cox proportional hazards regression model. The FR was calculated as follows: HR = hazard rate [H(t)] of the different categories/[H(t) reference category]. The main analyses are presented in eTable 7 in Supplement 1. Survival curves were derived from the unadjusted models.

Figure 1 shows that compared with couples without poverty, those experiencing poverty had lower fecundability (confounder model fecundability ratio [FR], 0.61 [95% CI, 0.51-0.72]). Compared with women with a high educational level, those with a low (FR, 0.61 [95% CI, 0.50-0.74]) and middle (FR, 0.68 [95% CI, 0.61-0.75]) educational level had lower fecundability. Similarly, compared with men with a high educational level, those with a low (FR, 0.72 [95% CI, 0.62-0.85]) and middle (FR, 0.72 [95% CI, 0.66-0.80]) educational level had lower fecundability. Compared with a household income of €6000 or more per month, a household income of less than €3000 per month was associated with lower fecundability (FR, 0.59 [95% CI, 0.51-0.68]). The results were largely similar after further adjustment (eTable 7 in Supplement 1). Sensitivity analyses that excluded women in the top 5% of the time-to-pregnancy observations did not materially change the effect estimates (eTable 8 in Supplement 1). Among Dutch participants only, low educational level of women and men was not associated with lower fecundability, possibly due to small sample sizes in these groups (eTable 9 in Supplement 1).

Table 2 shows that as compared with couples without poverty, those experiencing poverty had increased subfertility risk (32.5% vs 50.3%; relative risk [RR], 1.37 [95% CI, 1.16-1.62]). Also, as compared with high educational level, low educational level among women (31.9% vs 42.9%; RR, 1.30 [95% CI, 1.06-1.60]) and men (29.1% vs 38.8%; RR, 1.31 [95% CI, 1.10-1.57]) was associated with increased subfertility risk. When combining couples’ educational level, we observed that couples in which only 1 partner had a high educational level had increased subfertility risk compared with couples in which both partners had a high educational level. The highest risks were observed among couples in which neither partner had a high educational level (28.3% vs 38.7%; RR, 1.42 [95% CI, 1.23-1.64]. Table 2 also shows that as compared with a household income of €6000 or more per month, a household income of less than €3000 per month was associated with increased subfertility risk (30.4% vs 45.7%; RR, 1.47 [95% CI, 1.24-1.74]). These associations were only partly explained by demographic and lifestyle factors and remained significant in the fully adjusted models (eTable 10 in Supplement 1). The sensitivity analyses did not materially change the effect estimates (eTables 11-13 in Supplement 1).

**Table 2.** Associations of Poverty, Educational Level of Women and Men, and Household Income With Subfertility Risks^a^

Exposure	No.		Model, RR (95% CI)	
	Total	Subfertile (%)	Confounder	Fully adjusted
Poverty^b^	2628	883 (33.6)	NA	NA
Yes	169	85 (50.3)	1.37 (1.16-1.62)	1.11 (0.93-1.33)
No	2459	798 (32.5)	1 [Reference]	1 [Reference]
Educational level				
Women^c^	2770	957 (42.2)	NA	NA
Low	147	63 (42.9)	1.30 (1.06-1.60)	1.15 (0.94-1.41)
Middle	664	270 (40.7)	1.36 (1.21-1.52)	1.19 (1.05-1.35)
High	1959	624 (31.9)	1 [Reference]	1 [Reference]
Men^d^	2599	838 (32.2)	NA	NA
Low	232	90 (38.8)	1.31 (1.10-1.57)	1.26 (1.05-1.51)
Middle	785	288 (36.7)	1.31 (1.16-1.47)	1.27 (1.12-1.44)
High	1582	460 (29.1)	1 [Reference]	1 [Reference]
Women and men^e^	2599	838 (32.2)	NA	NA
Both without high level	584	226 (38.7)	1.42 (1.23-1.64)	1.29 (1.10-1.50)
Only women with high level	433	152 (35.1)	1.19 (1.03-1.39)	1.13 (0.97-1.32)
Only men with high level	148	54 (36.5)	1.31 (1.04-1.64)	1.17 (0.93-1.48)
Both with high level	1434	406 (28.3)	1 [Reference]	1 [Reference]
Household income per mo, €^f^	2717	933 (34.3)	NA	NA
<3000	658	301 (45.7)	1.47 (1.24-1.74)	1.15 (0.95-1.39)
3000-5999	1677	516 (30.8)	1.06 (0.90-1.24)	1.00 (0.85-1.18)
≥6000	382	116 (30.4)	1 [Reference]	1 [Reference]

Abbreviations: NA, not applicable; RR, relative risk.

^a^ The linearity assumption was tested for age at enrollment in all models; a significant quadratic term was found, which affected the estimates. Therefore, all models included a quadratic age term.

^b^ The confounder model was adjusted for participants’ age and parity. The fully adjusted model was adjusted for participants’ age, parity, migration background, cohabitation status, body mass index, alcohol consumption, smoking, and folic acid supplementation.

^c^ The confounder model was adjusted for participants’ age and parity. Parity was not significant. The fully adjusted model was adjusted for participants’ age, migration background, cohabitation status, body mass index, alcohol consumption, smoking, and folic acid supplementation.

^d^ The confounder model was adjusted for participants’ age. The fully adjusted model was adjusted for participants’ age, cohabitation status, body mass index, and smoking.

^e^ The confounder model was adjusted for age of women and men and parity. Parity was not significant. The fully adjusted model was adjusted for age of women and men, migration background of women, body mass index of women, alcohol consumption of women and men, and smoking of women and men.

^f^ The confounder model was adjusted for participant’s age and parity. Parity was not significant. The fully adjusted model was adjusted for participants’ age, migration background, cohabitation status, body mass index, alcohol consumption, smoking, and folic acid supplementation.

### Miscarriage

**Figure 2.**
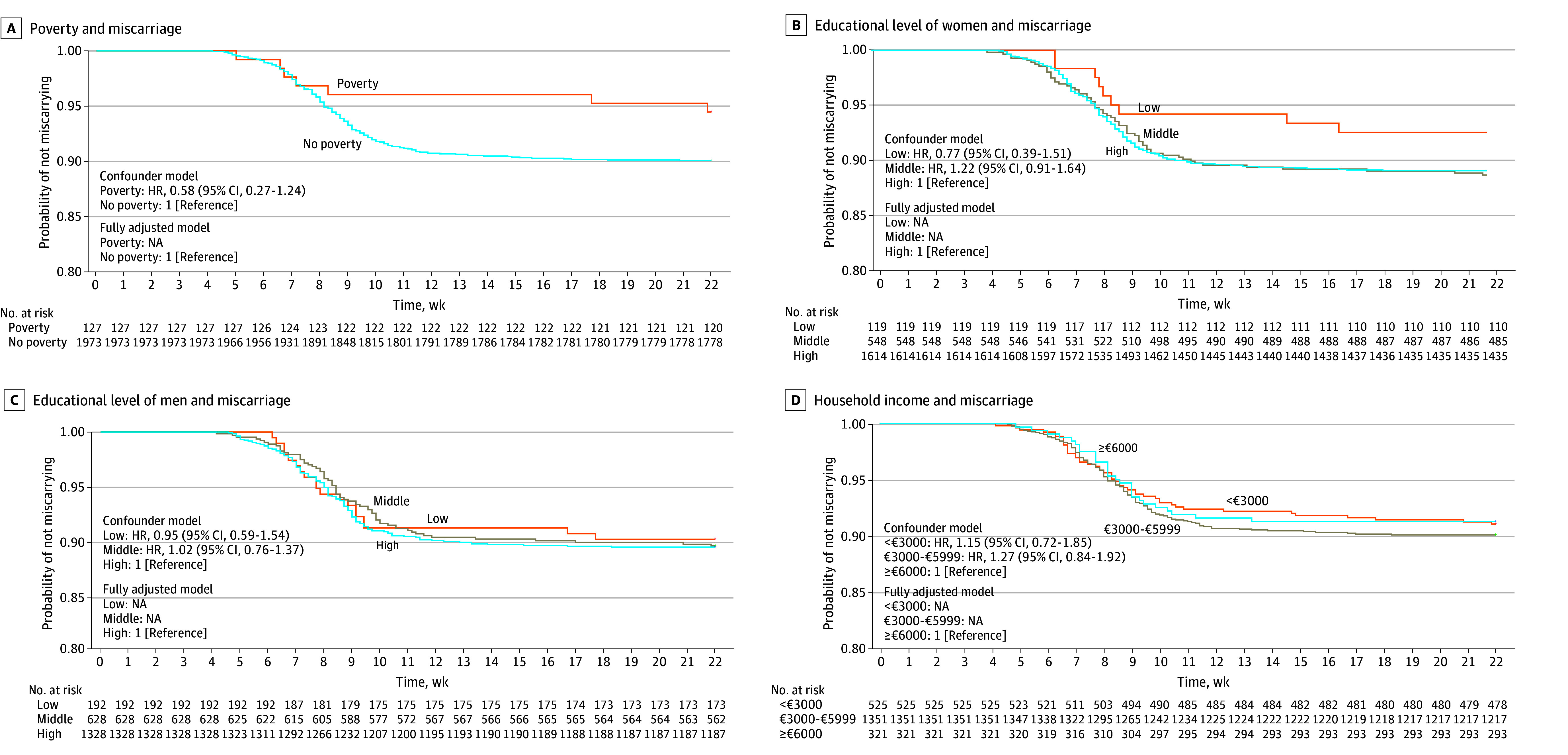
Associations of Poverty, Educational Level of Women and Men, and Household Income With Miscarriage Risk Hazard ratios (HRs) with 95% CIs of poverty (A), educational level (B and C), and household income (D) categories associated with miscarriage. HRs were derived from the Cox proportional hazards regression model. The HR was calculated as follows: HR = hazard rate [H(t)] of the different categories/[H(t) reference category]. The main analyses are presented in eTable 14 in Supplement 1. Survival curves were derived from the unadjusted models. NA indicates not applicable.

We observed no associations of poverty, educational level of women and men separately and combined, and household income with the probability of miscarriage per week of pregnancy (Figure 2). The results were largely similar after further adjustment (eTable 14 in Supplement 1). The sensitivity analyses did not materially change the effect estimates (eTables 15 and 16 in Supplement 1). Similarly, Table 3 shows that poverty, educational level, and household income were not associated with miscarriage risk. We did not further test any potential explanatory factors (eTable 16 in Supplement 1). The sensitivity analyses did not materially change the effect estimates (eTables 17-19 in Supplement 1).

**Table 3.** Associations of Poverty, Educational Level of Women and Men, and Household Income With Miscarriage Risks

Exposure	No.		Model, RR (95% CI)	
	Total	Miscarriage (%)	Confounder	Fully adjusted
Poverty^a^	2103	204 (9.7)	NA	NA
Yes	127	7 (5.5)	0.58 (0.28-1.19)	NA
No	1976	197 (10.0)	1 [Reference]	1 [Reference]
Educational level				
Women^a^	2285	254 (11.1)	NA	NA
Low	120	10 (8.3)	0.86 (0.47-1.57)	NA
Middle	549	64 (11.7)	1.23 (0.93-1.62)	NA
High	1616	180 (11.1)	1 [Reference]	1 [Reference]
Men^b^	2151	228 (10.6)	NA	NA
Low	192	19 (9.9)	0.94 (0.60-1.48)	NA
Middle	629	67 (10.7)	1.04 (0.79-1.37)	NA
High	1330	142 (10.7)	1 [Reference]	1 [Reference]
Women and men^c^	2151	228 (10.6)	NA	NA
Both without high level	478	50 (10.5)	1.22 (0.88-1.70)	NA
Only women with high level	343	36 (10.5)	1.02 (0.72-1.44)	NA
Only men with high level	121	16 (13.2)	1.34 (0.83-2.19)	NA
Both with high level	1209	126 (10.4)	1 [Reference]	1 [Reference]
Household income per mo, €^a^	2200	211 (9.6)	NA	NA
<3000	527	49 (9.3)	1.19 (0.77-1.84)	NA
3000-5999	1351	134 (9.9)	1.26 (0.86-1.86)	NA
≥6000	322	28 (8.7)	1 [Reference]	1 [Reference]

Abbreviations: NA, not applicable; RR, relative risk.

^a^ The confounder model was adjusted for participants’ age, parity, and history of miscarriage.

^b^ The confounder model was adjusted for participants’ age.

^c^ The confounder model was adjusted for age of women and men, parity, and history of miscarriage.

## Discussion

In this population-based prospective cohort study in an urban high-income setting from preconception onward, we observed that poverty and markers of social disadvantage were associated with lower fecundability and increased subfertility risks. These associations were only partly explained by demographic and lifestyle factors and showed the existence of a social gradient, in which greater social disadvantage was associated with progressively lower fecundability. Markers of social disadvantage were not associated with miscarriage risk. These results suggest that the origins of socioeconomic health disparities may lie in the earliest phases of life and specifically involve the preconception period during which gametogenesis occurs.^19,40,41,42^

In our study, the main exposures were poverty and measures of social disadvantage, including educational level and household income. Poverty was defined as having a household income of less than €3000 per month, in line with the Dutch poverty threshold, combined with the subjective measure of poverty, defined as not being able to pay for basic needs.^23,24^ Notably, all measures of social disadvantage were associated with lower fecundability, but poverty and household income were not associated with subfertility in the fully adjusted models, possibly due to limited statistical power. Furthermore, we observed a gradient effect of markers of social disadvantage. This indicates that social disadvantage across a wide range was associated with fertility.

Our results are in line with those of previous studies. A pregnancy cohort study among 1924 couples in the Netherlands reported that lower educational level was associated with lower fecundability.^43^ A preconception cohort among 10 475 women in Denmark observed that both lower educational level and household income were associated with lower fecundability.^15^ A preconception cohort among 8654 women in the US also observed that lower educational level was associated with lower fecundability.^17^ However, in contrast to our findings, the US study^17^ did not observe an association between household income and lower fecundability. Both of the previous preconception cohort studies^15,17^ used self-reported measures of time to pregnancy, including the LMP. A cohort study among 7472 postpartum women in Portugal reported that lower educational level among women was associated with higher odds of infertility.^44^ We also observed that men’s low educational level was independently associated with lower fecundability, even after adjusting for women’s educational level. This suggests that men’s socioeconomic position may also play a role in reproduction, possibly through pathways such as employment conditions, health literacy, and lifestyle behaviors that have been shown to influence sperm quality and fertility.^45,46,47^ However, in our study, effect estimates were only partly explained by demographic factors, including migration background and cohabitation status, or lifestyle factors, including body mass index, alcohol consumption, smoking, and folic acid supplementation. Currently unexplored pathways associated with social disadvantage, such as stress, health care access, neighborhood conditions, and cumulative disadvantage, may further explain these associations.

Social disadvantage may also be associated with miscarriage risk.^48,49^ A cohort study among 89 829 women in Denmark found that lower educational level and household income were associated with higher miscarriage risk.^50^ A prospective cohort study among 5806 women in Denmark observed no associations between women’s educational level and miscarriage risk, whereas a cohort study among 30 166 women in India observed that higher educational level among women was associated with increased miscarriage risk.^51,52^ A register-based study among 3 941 020 pregnancies in Asia observed that lower household income was associated with miscarriage risk.^53^ We observed no associations of poverty, educational level, or household income with miscarriage risk. This may partly be due to the relatively small numbers of miscarriages in subgroups with severe disadvantage and differences of miscarriage detection among countries.

The mechanisms by which socioeconomic disparities may affect reproductive outcomes are complex and multifactorial.^2,3,5,54,55^ The associations of poverty and social disadvantage with subfertility are likely multifactorial and cumulative, involving psychosocial, biological, and contextual factors.^56^ First, the findings of this study should be interpreted within the broader context of persistent health inequalities within high-income countries. Despite high-quality social services in high-income countries, socioeconomic gradients in health and health care access remain prevalent.^57,58^ Couples with social disadvantages may face barriers in navigating health care systems, understanding medical advice, and affording costs of assisted reproductive technology, resulting in lower fecundability and increased risks of subfertility.^16,59^ More specifically, a lower educational level can limit health literacy by challenging women and men to access, understand, and act upon reproductive health information properly.^60,61^ Second, women and men experiencing social disadvantage are more likely to experience barriers for engaging in and maintaining healthy behaviors, such as eating a balanced diet and avoiding smoking and excessive alcohol consumption.^62^ Financial constraints due to lower household income, lower health literacy, and living in environments with fewer healthy food options can contribute to an unhealthy diet.^63,64^ Social disadvantage could increase poor health behavior due to higher stress levels, reduced access to health information, and living in environments in which unhealthy coping behaviors are more prevalent.^65,66^ This may affect fertility through reduced metabolic health, hormonal disturbances, and impaired reproductive function. Third, lower educational level could result in employment with lower wages and unfavorable benefits. This increases financial strain and work-related stress, which could result in adverse reproductive health through hormonal dysfunction.^67^ A lower household income could also lead to more perceived financial barriers to use medical services.^68^ Finally, women and men experiencing poverty and having a lower household income are more prone to experience adverse life events such as divorce, job loss, and domestic violence, which further increase stress and subsequently may cause adverse maternal and child health outcomes.^69,70,71,72^ Yet despite proposed pathways, we acknowledge that other biological or medical factors could play a role. Future research should continue to investigate these pathways, including potential mediators such as stress, neighborhood disadvantage, and cumulative exposure to adverse conditions throughout the life course.

Our results highlight that even in urban high-income settings with good health care access, poverty and social disadvantage were associated with lower fecundability and increased subfertility risk. Addressing risk factors associated with social disadvantage could help reduce health inequality. This emphasizes the need for tailored preconception counseling that considers both partners’ social circumstances. By recognizing and addressing these broader social determinants, health care practitioners and policy-makers can better support couples who are trying to conceive, particularly those facing greater barriers as a result of poverty or lower educational level.

### Strengths and Limitations

This study’s strengths include its prospective study design, large sample size, use of different measures of social disadvantage, data collection at multiple time points, and the inclusion of men. That said, the study also has several limitations. First, generalizability may be limited. Despite broad recruitment efforts, voluntary participation may have led to underrepresentation of those experiencing social disadvantage, potentially underestimating the true effects on a population level. Second, not all data were collected prospectively. For women included during pregnancy, exposure data were obtained after conception. However, measures of social disadvantage are generally stable over time, reducing misclassification risk. Third, the date when couples actively pursued pregnancy was self-reported and possibly misinterpreted in our questionnaires, leading to misclassification. To account for this, we used both the date of actively pursuing pregnancy and the date of refraining from contraceptives. Fourth, recall bias may have affected accuracy of time to pregnancy and duration of actively pursuing pregnancy due to potentially retrospectively answered questionnaires. To address this, time to pregnancy was reconfirmed during the first trimester visit with the obstetric caregiver in which embryo size was measured, and pregnancy duration was estimated. Fifth, using months rather than menstrual cycles to estimate time to pregnancy may have introduced misclassification, particularly for women with irregular cycles. This could have led to inflated time-to-pregnancy estimates. However, subfertility is clinically defined by months, and this approach is common in population-based studies and supports translation of the results into clinical care.^30^ Sixth, early-pregnancy losses could have been misclassified as nonconceptions if they occurred before pregnancy recognition. This may have resulted in underestimating conception rates and early miscarriages. Finally, there are other factors that likely influence fertility and miscarriage risk, including diet stress, environmental exposures, medication, caffeine, physical activity, and broader contexts such as the COVID-19 pandemic. Stress, medication, and effects of broader contexts such as the COVID-19 pandemic were not fully captured in our questionnaires. We did not include caffeine or physical activity due to inconsistent evidence of their effects; instead, we focused on well-established explanatory factors to reduce measurement bias and improve interpretability. Although parental childhood conditions (eg, exposure to smoking, childhood obesity, and mental health disorders) may have long-term effects on adult reproductive health, our questionnaires addressed them only briefly and retrospectively, introducing recall bias and limiting our ability to adjust for them comprehensively. Diet was only measured in a small, preconceptionally included subgroup, so we were not able to include diet. Therefore, residual confounding remains possible, as in any observational study.

## Conclusions

In this population-based prospective cohort study from the preconception period onward, we observed that, even in a high-income country, poverty, low educational level, and household income were associated with lower fecundability and increased risks of subfertility but not with miscarriage risk. Acknowledging the effects of social factors in addition to biological factors associated with fertility and early-pregnancy outcomes could contribute to targeted and effective preventive strategies for couples desiring pregnancy.

## Notes

Supplement 1.
eMethods
**eFigure 1**. Description of Study Design by Inclusion Moment **eFigure 2.** Flowchart of Participants Included in the Time-to-Pregnancy Analyses **eFigure 3**. Flowchart of Participants Included in the Miscarriage Analyses **eTable 1.** Detailed Information of the Characteristics of the Subpopulations for Analyses **eTable 2.** Population Characteristics Presented per Level of Poverty **eTable 3.** Population Characteristics Presented per Level of Education Level of Women **eTable 4.** Population Characteristics Presented per Level of Education Level of Men **eTable 5.** Population Characteristics Presented per Level of Household Income **eTable 6.** Nonresponse Analysis of Participants Included and Excluded From the Study Populations **eTable 7.** Associations of Poverty, Educational Level of Women and Men, and Household Income With Fecundability Ratios **eTable 8.** Associations of Poverty, Educational Level of Women and Men, and Household Income With Fecundability Ratios, Excluding Top 5% of Time to Pregnancy **eTable 9.** Associations of Poverty, Educational Level of Women and Men, and Household Income With Fecundability Ratios, Including Only Dutch Participants **eTable 10.** Associations of Poverty, Educational Level of Women and Men, and Household Income With Risks of Subfertility **eTable 11.** Associations of Poverty, Educational Level of Women and Men, and Household Income With Risks of Subfertility, Excluding Couples Undergoing Assisted Reproductive Technology **eTable 12.** Associations of Poverty, Educational Level of Women and Men, and Household Income With Risks of Subfertility, Excluding Top 5% of Time to Pregnancy **eTable 13.** Associations of Poverty, Educational Level of Women and Men, and Household Income With Risks of Subfertility, Including Only Dutch Participants **eTable 14.** Associations of Poverty, Educational Level of Women and Men, and Household Income With Hazard Ratios of Miscarriage **eTable 15.** Associations of Poverty, Educational Level of Women and Men, and Household Income With Hazard Ratios of Miscarriage, Excluding Couples Undergoing Assisted Reproductive Technology **eTable 16.** Associations of Poverty, Educational Level of Women and Men, and Household Income With Hazard Ratios of Miscarriage, Including Only Dutch Participants **eTable 17.** Associations of Poverty, Educational Level of Women and Men, and Household Income With Risks of Miscarriage **eTable 18.** Associations of Poverty, Educational Level of Women and Men, and Household Income With Risks of Miscarriage, Excluding Couples Undergoing Assisted Reproductive Technology **eTable 19.** Associations of Poverty, Educational Level of Women and Men, and Household Income With Risks of Miscarriage, Including Only Dutch Participants

Supplement 2.
Data Sharing Statement
