# Supplementary material for: Poverty and Social Disadvantage in Women and Men and Fertility Outcomes
Source: JAMA Netw Open. 2025 Sep 19;8(9):e2532741. doi: 10.1001/jamanetworkopen.2025.32741 (PMC12449726; doi:10.1001/jamanetworkopen.2025.32741)
Supplement: Supplement 1. — eMethods eFigure 1. Description of Study Design by Inclusion Moment eFigure 2. Flowchart of Participants Included in the Time-to-Pregnancy Analyses eFigure 3. Flowchart of Participants Included in the Miscarriage Analyses eTable 1. Detailed Information of the Characteristics of the Subpopulations for Analyses eTable 2. Population Characteristics Presented per Level of Poverty eTable 3. Population Characteristics Presented per Level of Education Level of Women eTable 4. Population Characteristics Presented per Level of Education Level of Men eTable 5. Population Characteristics Presented per Level of Household Income eTable 6. Nonresponse Analysis of Participants Included and Excluded From the Study Populations eTable 7. Associations of Poverty, Educational Level of Women and Men, and Household Income With Fecundability Ratios eTable 8. Associations of Poverty, Educational Level of Women and Men, and Household Income With Fecundability Ratios, Excluding Top 5% of Time to Pregnancy eTable 9. Associations of Poverty, Educational Level of Women and Men, and Household Income With Fecundability Ratios, Including Only Dutch Participants eTable 10. Associations of Poverty, Educational Level of Women and Men, and Household Income With Risks of Subfertility eTable 11. Associations of Poverty, Educational Level of Women and Men, and Household Income With Risks of Subfertility, Excluding Couples Undergoing Assisted Reproductive Technology eTable 12. Associations of Poverty, Educational Level of Women and Men, and Household Income With Risks of Subfertility, Excluding Top 5% of Time to Pregnancy eTable 13. Associations of Poverty, Educational Level of Women and Men, and Household Income With Risks of Subfertility, Including Only Dutch Participants eTable 14. Associations of Poverty, Educational Level of Women and Men, and Household Income With Hazard Ratios of Miscarriage eTable 15. Associations of Poverty, Educational Level of Women and Men, and Household Income With Hazard Rat [file jamanetwopen-e2532741-s001.pdf]

Supplemental Online Content

Boxem AJ, Mulders AGMGJ, van Rossum I, Bekkers EL, Gaillard R, Jaddoe VWV. Poverty and social disadvantage in women and men and fertility outcomes. *JAMA Netw Open*. 2025;8(9):e2532741. doi:10.1001/jamanetworkopen.2025.32741

eMethods
eFigure 1. Description of Study Design by Inclusion Moment
eFigure 2. Flowchart of Participants Included in the Time-to-Pregnancy Analyses
eFigure 3. Flowchart of Participants Included in the Miscarriage Analyses
eTable 1. Detailed Information of the Characteristics of the Subpopulations for Analyses
eTable 2. Population Characteristics Presented per Level of Poverty
eTable 3. Population Characteristics Presented per Level of Education Level of Women
eTable 4. Population Characteristics Presented per Level of Education Level of Men
eTable 5. Population Characteristics Presented per Level of Household Income
eTable 6. Nonresponse Analysis of Participants Included and Excluded From the Study Populations
eTable 7. Associations of Poverty, Educational Level of Women and Men, and Household Income With Fecundability Ratios
eTable 8. Associations of Poverty, Educational Level of Women and Men, and Household Income With Fecundability Ratios, Excluding Top 5% of Time to Pregnancy
eTable 9. Associations of Poverty, Educational Level of Women and Men, and Household Income With Fecundability Ratios, Including Only Dutch Participants
eTable 10. Associations of Poverty, Educational Level of Women and Men, and Household Income With Risks of Subfertility
eTable 11. Associations of Poverty, Educational Level of Women and Men, and Household Income With Risks of Subfertility, Excluding Couples Undergoing Assisted Reproductive Technology
eTable 12. Associations of Poverty, Educational Level of Women and Men, and Household Income With Risks of Subfertility, Excluding Top 5% of Time to Pregnancy
eTable 13. Associations of Poverty, Educational Level of Women and Men, and Household Income With Risks of Subfertility, Including Only Dutch Participants
eTable 14. Associations of Poverty, Educational Level of Women and Men, and Household Income With Hazard Ratios of Miscarriage
eTable 15. Associations of Poverty, Educational Level of Women and Men, and Household Income With Hazard Ratios of Miscarriage, Excluding Couples Undergoing Assisted Reproductive Technology
eTable 16. Associations of Poverty, Educational Level of Women and Men, and Household Income With Hazard Ratios of Miscarriage, Including Only Dutch Participants
eTable 17. Associations of Poverty, Educational Level of Women and Men, and Household Income With Risks of Miscarriage
eTable 18. Associations of Poverty, Educational Level of Women and Men, and Household Income With Risks of Miscarriage, Excluding Couples Undergoing Assisted Reproductive Technology
eTable 19. Associations of Poverty, Educational Level of Women and Men, and Household Income With Risks of Miscarriage, Including Only Dutch Participants

This supplemental material has been provided by the authors to give readers additional information about their work.

eMethods

Calculation of Fecundability Ratio on the Categorical Scale

The Hazard Ratio (HR) of the exposure was calculated by dividing the hazard rate of conceiving within one month of the different categories of the exposures: poverty, educational level of women and men separate and combined, and household income, by the hazard rate of the reference category of the exposure:

HR = (H(t) ± exposure category) / (H(t) exposure reference category).

A HR < 1 indicates a lower fecundability as compared to the reference category of the exposure.

Definition of Educational Level

Educational level was defined based on the ISCED scores.

Category	ISCED level	ISCED definition
Low	0-2	- No education, primary education (primary school)<br>- Lower secondary education (middle school)
Middle	3-5	- Upper secondary education (high school, vocational training)<br>- Short-cycle tertiary education (associate degree)
High	6-8	- Higher education phase 1 (bachelor's degree or equivalent)<br>- Higher education phase 2 (master's degree or higher)

© 2025 Boxem AJ et al. *JAMA Network Open*.

eFigure 1. Description of Study Design by Inclusion Moment

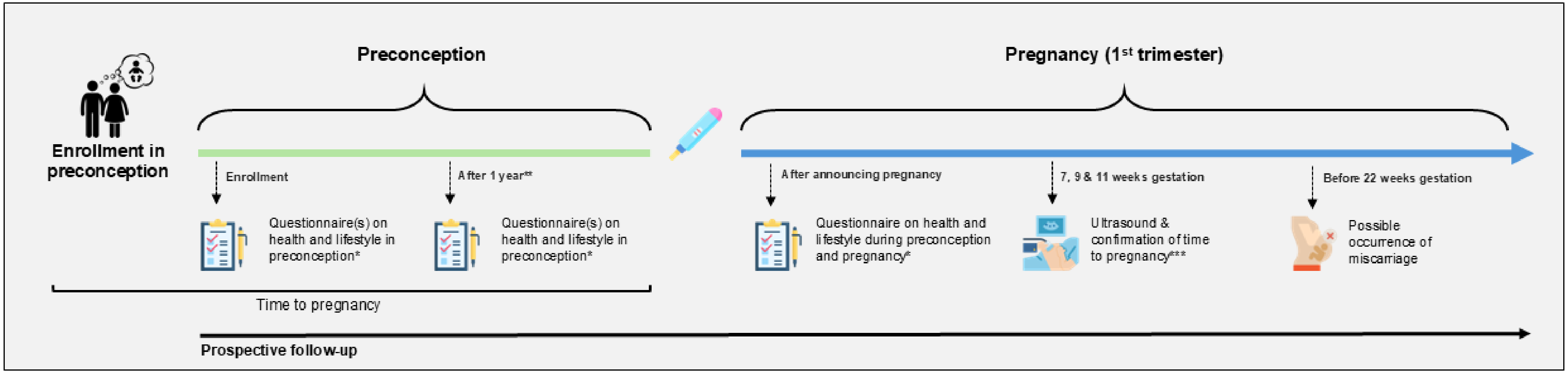

*Includes questions on the couple's age, ethnicity, educational level, income, alcohol use, drug use, smoking, (prepregnancy) weight and length, parity, supplement use, history of miscarriage, the first day of last menstrual period, the date of actively pursuing pregnancy and refraining from contraceptives

**After every subsequent year of preconception enrollment

***Time to pregnancy was obtained and reconfirmed in the prenatal questionnaire, through telephone contact when planning the first trimester visit(s), and based on embryonal size and due date confirmation during the dating ultrasound at the first trimester visit

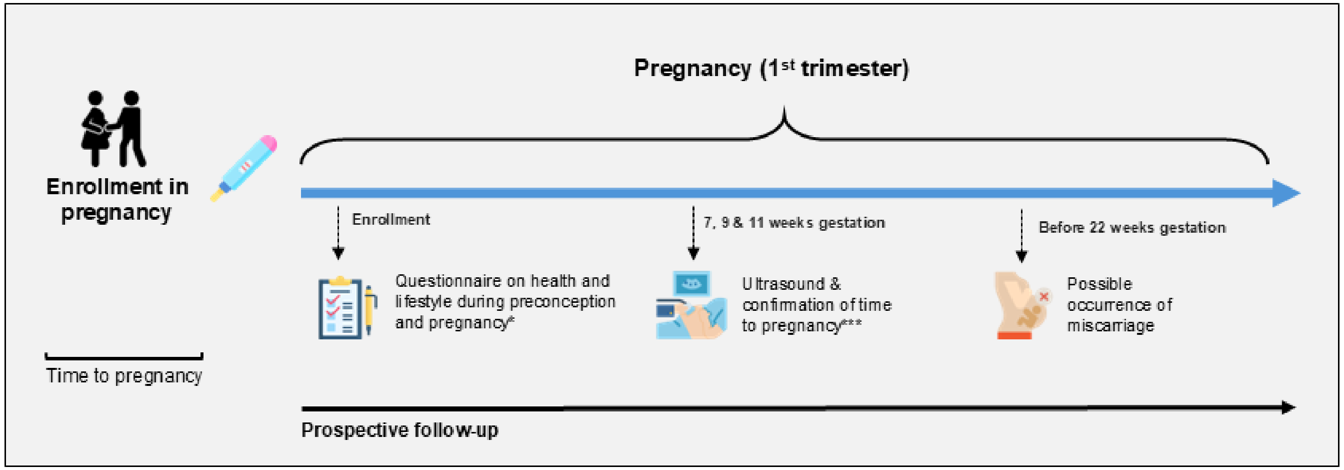

**Time to pregnancy analyses (preconceptional inclusion):**	Time to pregnancy is based on the ***preconceptionally*** reported date of pursuing pregnancy, and the first day of the last menstrual period reported during confirmation of the pregnancy to the research center when planning the first trimester visit(s). This date was reconfirmed in the prenatal questionnaire and during the dating ultrasound, based on embryo size and due date.
**Time to pregnancy analyses (prenatal inclusion):**	Time to pregnancy is based on the ***prenatally*** reported date of pursuing pregnancy, and the first day of the last menstrual period reported during confirmation of the pregnancy to the research center when planning the first trimester visit(s). This date was reconfirmed in the prenatal questionnaire and during the dating ultrasound, based on embryo size and due date.
**Miscarriage analyses:**	Prospective assessment from self-performed positive pregnancy test onwards. Selection bias may occur due to occurrence of miscarriages before inclusion or unrecognized miscarriages.

Icons designed by Flaticon (www.flaticon.com)

© 2025 Boxem AJ et al. *JAMA Network Open*.

eFigure 2. Flowchart of Participants Included in the Time-to-Pregnancy Analyses

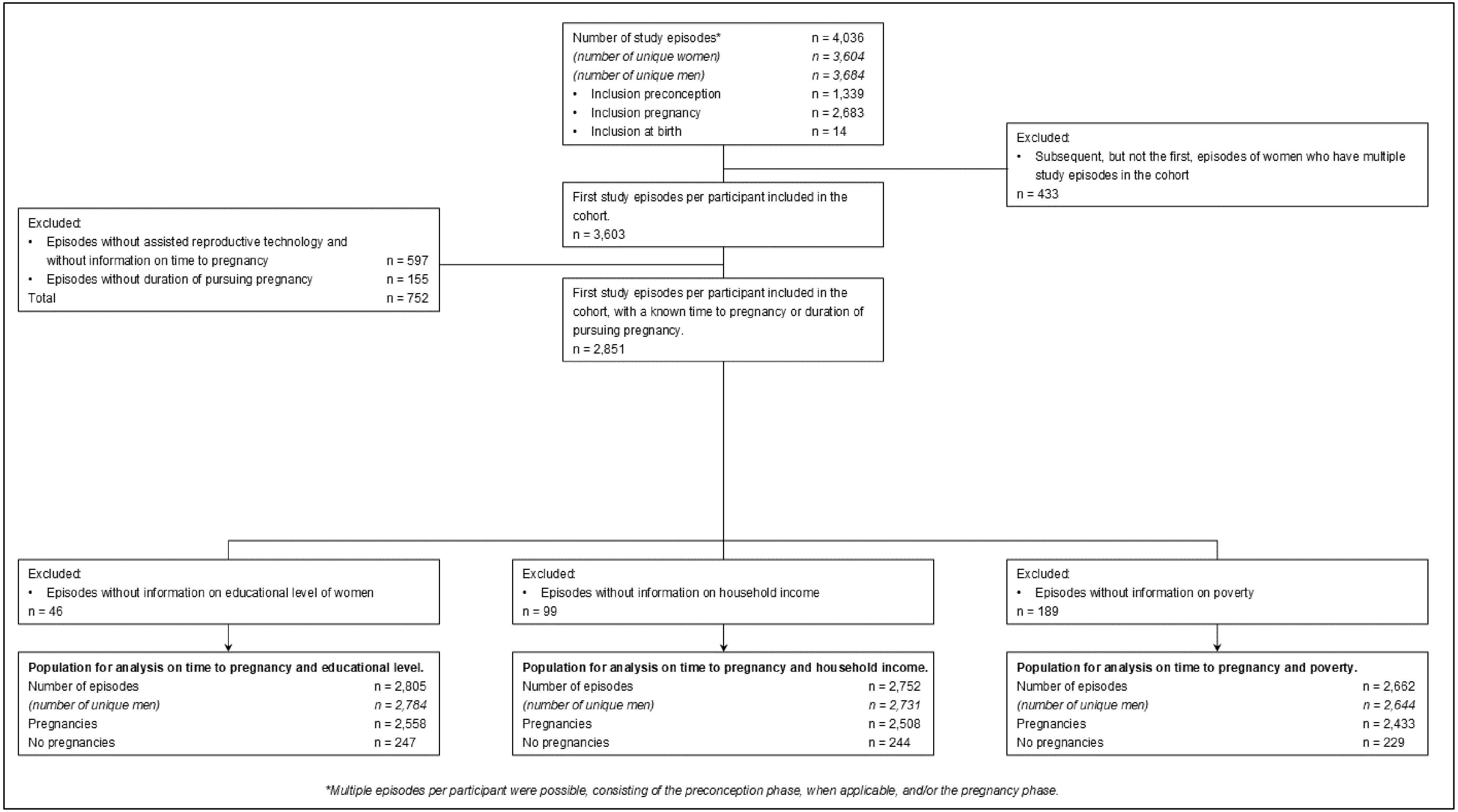

© 2025 Boxem AJ et al. *JAMA Network Open*.

eFigure 3. Flowchart of Participants Included in the Miscarriage Analyses

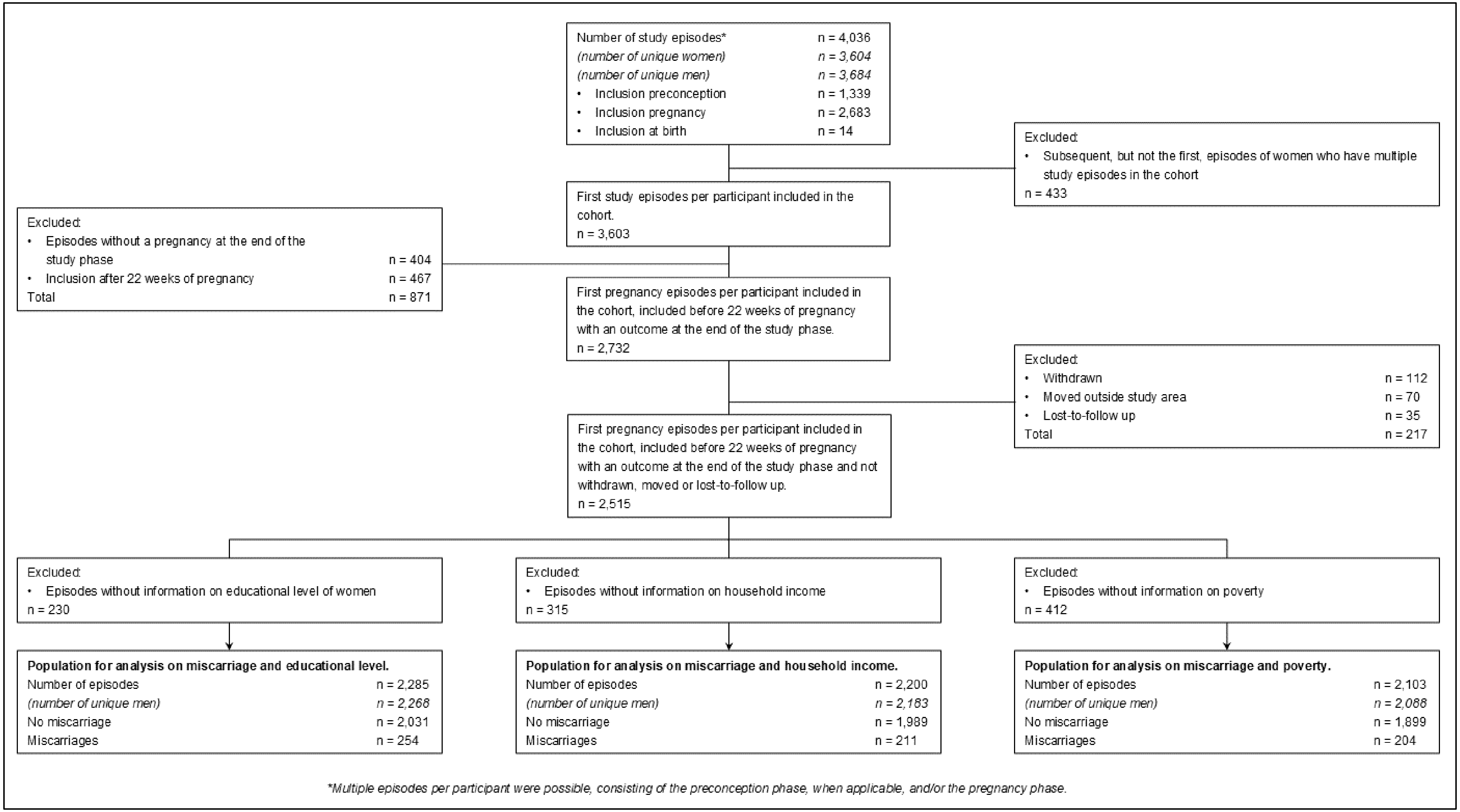

© 2025 Boxem AJ et al. *JAMA Network Open*.

eTable 1. Detailed Information of the Characteristics of the Subpopulations for Analyses

	Participants [a], No. (%)							
	Educational level				Household income		Poverty	
	Time to Pregnancy		Miscarriage		Time to Pregnancy	Miscarriage	Time to Pregnancy	Miscarriage
	Women	Men	Women	Men				
Characteristic	N=2,805	N=2,633	N=2,285	N=2,151	N=2,752	N=2,200	N=2,662	N=2,103
Age, median [IQR], y	31.5 [29.1, 34.3]	33.3 [30.4, 36.6]	31.4 [29.0, 34.1]	33.3 [30.3, 36.6]	31.5 [29.2, 34.4]	31.4 [29.0, 34.1]	31.6 [29.3, 34.3]	31.5 [29.2, 34.1]
Missing	*0*	*491 (18.6)*	*0*	*227 (10.6)*	*0*	*0*	*0*	*0*
Migration background								
Dutch	1716 (61.4)	1594 (62.5)	1422 (62.5)	1326 (62.9)	1686 (61.5)	1384 (63.2)	1659 (62.6)	1355 (64.7)
European [b]	285 (10.2)	216 (8.5)	220 (9.7)	164 (7.8)	280 (10.2)	211 (9.6)	269 (10.1)	204 (9.7)
Non-European [c]	792 (28.4)	741 (29.0)	634 (27.9)	619 (29.4)	775 (28.3)	596 (27.2)	723 (27.3)	536 (25.6)
Missing	*12 (0.4)*	*82 (3.1)*	*9 (0.4)*	*42 (2.0)*	*11 (0.4)*	*9 (0.4)*	*11 (0.4)*	*8 (0.4)*
Poverty [d]								
Yes	165 (6.3)	124 (5.0) [j]	124 (6.0)	93 (4.7) [j]	169 (6.3)	127 (6.0)	169 (6.3)	127 (6.0)
No	2467 (93.7)	2361 (95.0) [j]	1953 (94.0)	1881 (95.3) [j]	2493 (93.7)	1976 (94.0)	2493 (93.7)	1976 (94.0)
Missing	*173 (6.2)*	*148 (5.6)* [j]	*208 (9.1)*	*177 (8.2)* [j]	*90 (3.3)*	*97 (4.4)*	*0*	*0*
Educational level [e]								
Low	147 (5.2)	234 (8.9)	120 (5.3)	192 (8.9)	138 (5.1)	113 (5.2)	126 (4.8)	93 (4.5)
Middle	669 (23.9)	794 (30.2)	549 (24.0)	629 (29.2)	640 (23.5)	507 (23.3)	604 (22.9)	468 (22.5)
High	1989 (70.9)	1605 (61.0)	1616 (70.7)	1330 (61.8)	1944 (71.4)	1554 (71.5)	1902 (72.3)	1516 (73.0)
Missing	*0*	*0*	*0*	*0*	*30 (1.1)*	*26 (1.2)*	*30 (1.1)*	*26 (1.2)*
Household income in euros per month [f]								
Less than 3,000	654 (24.0)	531 (20.8) [j]	519 (23.9)	428 (20.8) [j]	664 (24.1)	527 (24.0)	621 (23.3)	467 (22.2)
3,000-5,999	1681 (61.8)	1642 (64.2) [j]	1337 (61.5)	1314 (63.9) [j]	1697 (61.7)	1351 (61.4)	1662 (62.4)	1326 (63.1)
Equal or more than 6,000	387 (14.2)	383 (15.0) [j]	318 (14.6)	313 (15.2) [j]	391 (14.2)	322 (14.6)	379 (14.2)	310 (14.7)
Missing	*83 (3.0)*	*77 (2.9)* [j]	*111 (4.9)*	*96 (4.5)* [j]	*0*	*0*	*0*	*0*
Cohabitation status [g]								
Not cohabiting	325 (12.2)	216 (8.6) [j]	240 (11.4)	169 (8.5) [j]	319 (12.2)	234 (11.3)	316 (12.2)	231 (11.2)

© 2025 Boxem AJ et al. *JAMA Network Open*.

	Participants [a], No. (%)							
	Educational level				Household income		Poverty	
	Time to Pregnancy		Miscarriage		Time to Pregnancy	Miscarriage	Time to Pregnancy	Miscarriage
	Women	Men	Women	Men				
Characteristic	N=2,805	N=2,633	N=2,285	N=2,151	N=2,752	N=2,200	N=2,662	N=2,103
Cohabiting	2338 (87.8)	2301 (91.4) [j]	1859 (88.6)	1827 (91.5) [j]	2297 (87.8)	1833 (88.7)	2284 (87.8)	1823 (88.8)
Missing	*142 5.1*	*116 4.4* [j]	*186 8.1*	*155 7.2* [j]	*136 4.9*	*133 6.0*	*62 2.3*	*49 2.3*
BMI, median [IQR], kg/m^2	23.4 [21.2, 26.5]	24.9 [23.0, 27.4]	23.4 [21.2, 26.4]	24.9 [23.0, 27.4]	23.4 [21.2, 26.5]	23.3 [21.1, 26.3]	23.4 [21.2, 26.4]	23.3 [21.1, 26.2]
Missing	*34 (1.2)*	*579 (22.0)*	*22 (1.0)*	*319 (14.8)*	*31 (1.1)*	*12 (0.5)*	*13 (0.5)*	*5 (0.2)*
Smoking								
No	1522 (55.6)	1276 (52.7)	1211 (56.0)	1001 (50.4)	1502 (55.9)	1192 (56.0)	1494 (56.2)	1184 (56.5)
No, quit smoking before pregnancy	847 (31.0)	553 (22.8)	644 (29.8)	483 (24.3)	824 (30.7)	627 (29.5)	817 (30.8)	620 (29.6)
Yes, smoked during pregnancy	366 (13.4)	593 (24.5)	308 (14.2)	503 (25.3)	360 (13.4)	309 (14.5)	345 (13.0)	292 (13.9)
Missing	*70 (2.5)*	*211 (8.0)*	*122 (5.3)*	*164 (7.6)*	*66 (2.4)*	*72 (3.3)*	*6 (0.2)*	*7 (0.3)*
Alcohol consumption								
No consumption < 3 months before pregnancy	580 (21.2)	330 (13.0)	463 (21.3)	251 (12.4)	564 (21.0)	451 (21.1)	545 (20.6)	435 (20.7)
Yes, consumption < 3 months before pregnancy	1761 (64.4)	2218 (87.0)	1340 (61.7)	1770 (87.6)	1737 (64.7)	1326 (62.1)	1727 (65.2)	1314 (62.6)
Yes, consumption during pregnancy	394 (14.4)	NA	368 (17.0)	NA	384 (14.3)	359 (16.8)	376 (14.2)	349 (16.6)
Missing	*70 (2.5)*	*85 (3.2)*	*114 (5.0)*	*130 (6.0)*	*67 (2.4)*	*64 (2.9)*	*14 (0.5)*	*5 (0.2)*
Folic acid supplementation								
Never	26 (1.0)	22 (0.9) [j]	20 (1.0)	18 (0.9) [j]	26 (1.0)	20 (1.0)	26 (1.0)	20 (1.0)
Started prior to pregnancy	1749 (67.8)	1674 (68.2) [j]	1403 (67.0)	1349 (67.8) [j]	1721 (67.9)	1382 (67.0)	1712 (67.9)	1375 (67.1)
Started in pregnancy	806 (31.2)	759 (30.9) [j]	671 (32.0)	624 (31.3) [j]	787 (31.1)	660 (32.0)	782 (31.0)	654 (31.9)
Missing	*224 (8.0)*	*178 (6.8)* [j]	*191 (8.4)*	*160 (7.4)* [j]	*218 (7.9)*	*138 (6.3)*	*142 (5.3)*	*54 (2.6)*
Parity								
Nulliparous	1851 (68.0)	1747 (68.1) [j]	1465 (67.2)	1393 (67.5) [j]	1819 (68.0)	1429 (67.6)	1810 (68.1)	1419 (67.6)
Multiparous	870 (32.0)	819 (31.9) [j]	716 (32.8)	671 (32.5) [j]	855 (32.0)	684 (32.4)	848 (31.9)	679 (32.4)

© 2025 Boxem AJ et al. *JAMA Network Open*.

	Participants [a], No. (%)							
	**Educational level**				**Household income**		**Poverty**	
	**Time to Pregnancy**		**Miscarriage**		**Time to Pregnancy**	**Miscarriage**	**Time to Pregnancy**	**Miscarriage**
	**Women**	**Men**	**Women**	**Men**			**Women**	**Men**
Characteristic	N=2,805	N=2,633	N=2,285	N=2,151	N=2,752	N=2,200	N=2,805	N=2,633
Missing	*84 (3.0)*	*67 (2.5)* [j]	*104 (4.6)*	*87 (4.0)* [j]	*78 (2.8)*	*87 (4.0)*	*4 (0.2)*	*5 (0.2)*
Miscarriage in previous pregnancy								
No	2200 (80.6)	2078 (80.8) [j]	1749 (81.3)	1661 (81.4) [j]	2166 (80.9)	1724 (81.5)	2156 (81.0)	1717 (81.6)
Yes	528 (19.4)	494 (19.2) [j]	402 (18.7)	379 (18.6) [j]	513 (19.1)	392 (18.5)	506 (19.0)	386 (18.4)
Missing	*77 (2.7)*	*61 (2.3)* [j]	*134 (5.9)*	*111 (5.2)* [j]	*73 (2.7)*	*84 (3.8)*	*0*	*0*
Time to pregnancy, median *(95% range)* [h]	3.5 [0.0, 67.8]	3.4[0.0, 62.1] [j]	3.3 [0.0, 61.4]	3.2 [0.0, 54.9] [j]	3.4 [0.0, 67.2]	3.3 [0.0, 60.4]	3.4 [0.0, 65.5]	3.2 [0.0, 63.8]
0-12 months	1813 (64.6)	1761 (66.9) [j]	1519 (72.2)	1479 (73.7) [j]	1784 (64.8)	1500 (72.4)	1745 (65.6)	1473 (72.8)
> 12 months	469 (16.7)	429 (16.3) [j]	358 (17.0)	333 (16.6) [j]	455 (16.5)	349 (16.8)	434 (16.3)	335 (16.6)
ART leading to pregnancy	276 (9.8)	237 (9.0) [j]	227 (10.8)	195 (9.7) [j]	269 (9.8)	223 (10.8)	254 (9.5)	215 (10.6)
Not pregnant	247 (8.8)	206 (7.8) [j]	NA	NA [j]	244 (8.9)	NA	229 (8.6)	NA
Overall subfertility [i]	957 (34.5)	838 (32.2) [j]	585 (27.8)	528 (26.3) [j]	933 (34.3)	572 (27.6)	883 (33.6)	550 (27.2)
Missing	*0*	*0* [j]	*181 (7.9)*	*144 (6.7)* [j]	*0*	*128 (5.8)*	*0*	*80 (3.8)*
Occurrence of miscarriage								
No miscarriage	2355 (92.1)	2243 (92.4) [j]	2031 (88.9)	1923 (89.4) [j]	2308 (92.0)	1989 (90.4)	2237 (91.9)	1899 (90.3)
Miscarriage	203 (7.9)	184 (7.6) [j]	254 (11.1)	228 (10.6) [j]	200 (8.0)	211 (9.6)	196 (8.1)	204 (9.7)
Missing	*247 (8.8)*	*206 (7.8)* [j]	*0*	*0* [j]	*244 (8.9)*	*0*	*229 (8.6)*	*0*
Timing of miscarriage, median [IQR], wk.	8.1 [7.0, 9.6]	8.1 [7.0, 9.5] [j]	8.14 [7.00, 9.43]	8.1 [7.0, 9.3] [j]	8.1 [7.0, 9.6]	8.1 [7.0, 9.6]	8.1 [7.0, 9.5]	8.1 [7.0, 9.5]
First trimester	187 (93.0)	170 (93.4) [j]	236 (94.0)	213 (94.2) [j]	184 (92.9)	195 (93.3)	181 (93.3)	189 (93.6)
Second trimester	14 (7.0)	12 (6.6) [j]	15 (6.0)	13 (5.8) [j]	14 (7.1)	14 (6.7)	13 (6.7)	13 (6.4)
Missing	*2 (1.0)*	*2 (1.1)* [j]	*3 (1.2)*	*2 (0.9)* [j]	*2 (1.0)*	*2 (0.9)*	*2 (1.0)*	*2 (1.0)*

Abbreviations: ART, assisted reproductive technology; BMI, body mass index (calculated as weight in kilograms divided by height in meters squared); NA, not applicable.

a. Total study population consisting of 3,604 unique women from Rotterdam, the Netherlands, with a total of 4,036 participant episodes leading to 3,577 pregnancy episodes. Women were included in preconception and pregnancy between 2017 and 2021.
b. Included: European, German, Yugoslav, or Polish migration background.
c. Included: African; American, non-western; Asian, non-western; Chinese; Indonesian; American, western; Asian, western; Cape Verdean; Dutch Antilles; Moroccan; Oceanian; Surinamese, or Turkish migration background.

© 2025 Boxem AJ et al. *JAMA Network Open*.

d. ‘Yes’ consists of ‘experiencing financial difficulties’ combined with ‘household income of less than 3000 euros per month’. ‘No’ consists of ‘experiencing financial difficulties’ combined with ‘household income of 3,000-5,999 euros per month’ and ‘equal or more than 6,000 euros per month’ as well as ‘not experiencing financial difficulties’ combined with ‘household income of ‘less than 3,000 euros per month’, ‘3,000-5,999 euros per month’ and ‘equal or more than 6,000 euros per month’.
e. ‘Low’ consists of ‘no primary education finished’, ‘primary education finished’ and ‘secondary education phase 1 finished’, ‘middle’ consists of ‘secondary education phase 2 finished’ and ‘high’ consists of ‘higher education phase 1 finished’ and ‘higher education phase 2 finished’.
f. ‘Less than 3,000 euros per month’ consists of ‘less than 900 euros per month’, ‘1,000-1,999 euros per month’ and ‘2,000-2,999 euros per month’. ‘3,000-5,999 euros per month’ consists of ‘3,000-3,999 euros per month’, ‘4,000-4,999 euros per month’ and ‘5,000-5,999 euros per month’.
g. ‘Not cohabiting’ consisted of ‘no partner and/or not cohabiting’ and ‘married, living separately’. ‘Cohabiting’ consisted of ‘cohabiting, but not married’, ‘married and cohabiting with spouse’, and ‘registered partnership and cohabiting’.
h. Time to pregnancy in months was derived from pregnancy episodes with a natural conception.
i. For the educational level analyses: episodes with ART leading to pregnancy, episodes without pregnancy and use of ART (7 of 247episodes), and episodes without pregnancy, without use of ART, and a duration of actively pursuing pregnancy of more than 12 months (205 of 247episodes) were added to the subfertile group (time to pregnancy > 12 months and use of ART) in the analysis. For the income analyses: episodes with ART leading to pregnancy, episodes without pregnancy and use of ART (7 of 244episodes), and episodes without pregnancy, without use of ART and a duration of actively pursuing pregnancy of more than 12 months (202 of 244episodes) were added to the subfertile group (time to pregnancy > 12 months and use of ART) in the analysis. For the poverty analyses: episodes with ART leading to pregnancy, episodes without pregnancy and use of ART (7 of 229episodes), and episodes without pregnancy, without use of ART and a duration of actively pursuing pregnancy of more than 12 months (188 of 229episodes) were added to the subfertile group (time to pregnancy > 12 months and use of ART) in the analysis.
j. Household income, poverty, cohabitation status, folic acid supplementation, parity, miscarriage in previous pregnancy, time to pregnancy in months, occurrence of miscarriage, timing of miscarriage in weeks of men were derived from their partner.

© 2025 Boxem AJ et al. *JAMA Network Open*.

eTable 2. Population Characteristics Presented per Level of Poverty

	Episodes, No. (%)					
	Poverty					
	Time to pregnancy N=2,662			Miscarriage N=2,103		
	Yes	No	p-value	Yes	No	p-value
Characteristic	N=169	N=2,493		N=127	N=1,976	
Age, median [IQR], y	31.2 [26.5, 35.5]	31.6 [29.4, 34.3]	0.147	30.9 [26.2, 34.7]	31.5 [29.3, 34.1]	0.021
Missing	*0*	*0*		*0*	*0*	
Migration background						
Dutch	46 (27.7)	1613 (64.9)	<0.001	40 (31.5)	1315 (66.8)	<0.001
European [a]	15 (9.0)	254 (10.2)		14 (11.0)	190 (9.7)	
Non-European [b]	105 (63.3)	618 (24.9)		73 (57.5)	463 (23.5)	
Missing	*3 (1.8)*	*8 (0.3)*		*0*	*8 (0.4)*	
Educational level [c]						
Low	31 (18.8)	95 (3.9)	<0.001	24 (19.4)	69 (3.5)	<0.001
Middle	92 (55.8)	512 (20.8)		70 (56.5)	398 (20.4)	
High	42 (25.5)	1860 (75.4)		30 (24.2)	1486 (76.1)	
Missing	*4 (2.4)*	*26 (1.0)*		*3 (2.4)*	*23 (1.2)*	
Household income in euros per month [d]						
Less than 3,000	169 (100.0)	452 (18.1)	<0.001	127 (100.0)	340 (17.2)	<0.001
3,000-5,999	0 (0.0)	1662 (66.7)		0 (0.0)	1326 (67.1)	
Equal or more than 6,000	0 (0.0)	379 (15.2)		0 (0.0)	310 (15.7)	
Missing	*0*	*0*		*0*	*0*	
Cohabitation status [e]						
Not cohabiting	81 (50.6)	235 (9.6)	<0.001	65 (53.7)	166 (8.6)	<0.001
Cohabiting	79 (49.4)	2205 (90.4)		56 (46.3)	1767 (91.4)	
Missing	*9 (5.3)*	*53 (2.1)*		*6 (4.7)*	*43 (2.2)*	
BMI, median [IQR], kg/m^2	26.7 [23.2, 30.7]	23.2 [21.1, 26.2]	<0.001	26.6 [23.1, 30.0]	23.1 [21.1, 25.9]	<0.001
Missing	*2 (1.2)*	*11 (0.4)*		*1 (0.8)*	*4 (0.2)*	

© 2025 Boxem AJ et al. *JAMA Network Open*.

	Episodes, No. (%)					
	Poverty					
	Time to pregnancy N=2,662			Miscarriage N=2,103		
	Yes	No	p-value	Yes	No	p-value
Characteristic	N=169	N=2,493		N=127	N=1,976	
Smoking						
No	62 (36.9)	1432 (57.6)	<0.001	42 (33.6)	1142 (57.9)	<0.001
No, quit smoking before pregnancy	40 (23.8)	777 (31.2)		23 (18.4)	597 (30.3)	
Yes, smoked during pregnancy	66 (39.3)	279 (11.2)		60 (48.0)	232 (11.8)	
Missing	*1 (0.6)*	*5 (0.2)*		*2 (1.6)*	*5 (0.3)*	
Alcohol consumption						
No consumption < 3 months before pregnancy	64 (38.1)	481 (19.4)	<0.001	53 (41.7)	382 (19.4)	<0.001
Yes, consumption < 3 months before pregnancy	82 (48.8)	1645 (66.3)		54 (42.5)	1260 (63.9)	
Yes, consumption during pregnancy	22 (13.1)	354 (14.3)		20 (15.7)	329 (16.7)	
Missing	*1 (0.6)*	*13 (0.5)*		*0*	*5 (0.3)*	
Folic acid supplementation						
Never	11 (7.5)	15 (0.6)	<0.001	6 (5.1)	14 (0.7)	<0.001
Started prior to pregnancy	46 (31.3)	1666 (70.2)		36 (30.5)	1339 (69.3)	
Started in pregnancy	90 (61.2)	692 (29.2)		76 (64.4)	578 (29.9)	
Missing	*22 (13.0)*	*120 (4.8)*		*9 (7.1)*	*45 (2.3)*	
Parity						
Nulliparous	81 (47.9)	1729 (69.5)	<0.001	62 (48.8)	1357 (68.8)	<0.001
Multiparous	88 (52.1)	760 (30.5)		65 (51.2)	614 (31.2)	
Missing	*0*	*4 (0.2)*		*0*	*5 (0.3)*	
Miscarriage in previous pregnancy						
No	121 (71.6)	2035 (81.6)	0.002	96 (75.6)	1621 (82.0)	0.089

© 2025 Boxem AJ et al. *JAMA Network Open*.

	Episodes, No. (%)					
	Poverty					
	Time to pregnancy N=2,662			Miscarriage N=2,103		
	Yes	No	p-value	Yes	No	p-value
Characteristic	N=169	N=2,493		N=127	N=1,976	
Yes	48 (28.4)	458 (18.4)		31 (24.4)	355 (18.0)	
Missing	*0*	*0*		*0*	*0*	
Time to pregnancy, median *(95% range)* [f]	6.7 [0.0, 135.9]	3.2 [0.0, 57.6]	<0.001	6.1 [0.0, 141.6]	3.1 [0.0, 51.7]	<0.001
0-12 months	84 (49.7)	1661 (66.6)	<0.001	63 (57.8)	1410 (73.7)	<0.001
> 12 months	56 (33.1)	378 (15.2)		37 (33.9)	298 (15.6)	
ART leading to pregnancy	11 (6.5)	243 (9.7)		9 (8.3)	206 (10.8)	
Not pregnant	18 (10.7)	211 (8.5)		NA	NA	
Overall subfertility [g]	85 (50.3)	798 (32.5)		46 (42.2)	504 (26.3)	
Missing	*0*	*0*		*18 (14.2)*	*62 (3.1)*	
Occurrence of miscarriage						
No miscarriage	144 (95.4)	2093 (91.7)	0.150	120 (94.5)	1779 (90.0)	0.136
Miscarriage	7 (4.6)	189 (8.3)		7 (5.5)	197 (10.0)	
Missing	*18 (10.7)*	*211 (8.5)*		*0*	*0*	
Timing of miscarriage, median [IQR], wk.	7.1 [6.6, 13.0]	8.1 [7.0, 9.5]	0.656	7.1 [6.6, 13.0]	8.1 [7.0, 9.5]	0.671
First trimester	5 (71.4)	176 (94.1)		5 (71.4)	184 (94.4)	0.100
Second trimester	2 (28.6)	11 (5.9)		2 (28.6)	11 (5.6)	
Missing	*0*	*2 (1.1)*		*0*	*2 (1.0)*	

Abbreviations: ART, assisted reproductive technology; BMI, body mass index (calculated as weight in kilograms divided by height in meters squared); NA, not applicable.

a. Included: European, German, Yugoslav, or Polish migration background.
b. Included: African; American, non-western; Asian, non-western; Chinese; Indonesian; American, western; Asian, western; Cape Verdean; Dutch Antilles; Moroccan; Oceanian; Surinamese, or Turkish migration background.
c. 'Low' consists of 'no primary education finished', 'primary education finished' and 'secondary education phase 1 finished', 'middle' consists of 'secondary education phase 2 finished' and 'high' consists of 'higher education phase 1 finished' and 'higher education phase 2 finished'.
d. 'Less than 3,000 euros per month' consists of 'less than 900 euros per month', '1,000-1,999 euros per month' and '2,000-2,999 euros per month'. '3,000-5,999 euros per month' consists of '3,000-3,999 euros per month', '4,000-4,999 euros per month' and '5,000-5,999 euros per month'.
e. 'Not cohabiting' consisted of 'no partner and/or not cohabiting' and 'married, living separately'. 'Cohabiting' consisted of 'cohabiting, but not married', 'married and cohabiting with spouse', and 'registered partnership and cohabiting'.
f. Time to pregnancy in months was derived from pregnancy episodes with a natural conception.

© 2025 Boxem AJ et al. *JAMA Network Open*.

g. For poverty: episodes with ART leading to pregnancy, episodes without pregnancy and use of ART (0 of 18 episodes), and episodes without pregnancy, without use of ART, and a duration of actively pursuing pregnancy of more than 12 months (18 of 18 episodes) were added to the subfertile group (time to pregnancy > 12 months and use of ART) in the analysis. For no poverty: episodes with ART leading to pregnancy, episodes without pregnancy and use of ART (7 of 211 episodes), and episodes without pregnancy, without use of ART and a duration of actively pursuing pregnancy of more than 12 months (170 of 211 episodes) were added to the subfertile group (time to pregnancy > 12 months and use of ART) in the analysis.

© 2025 Boxem AJ et al. *JAMA Network Open*.

eTable 3. Population Characteristics Presented per Level of Education Level of Women

	Participants, No. (%)							
	Women							
	Educational level							
	Time to pregnancy N=2,805				Miscarriage N=2,285			
	**Low**	**Middle**	**High**	**p-value**	**Low**	**Middle**	**High**	**p-value**
Characteristic	N=147	N=669	N=1,989		N=120	N=549	N=1,616	
Age, median [IQR], y	30.5 [25.9, 34.2]	29.7 [26.6, 33.4]	32.0 [29.9, 34.7]	<0.001	30.3 [25.5, 33.9]	29.5 [26.3, 33.2]	31.8 [29.9, 34.4]	<0.001
Missing	*0*	*0*	*0*		*0*	*0*	*0*	
Migration background								
Dutch	69 (47.3)	356 (53.6)	1291 (65.1)	<0.001	61 (50.8)	290 (53.1)	1071 (66.5)	<0.001
European [a]	17 (11.6)	47 (7.1)	221 (11.1)		12 (10.0)	34 (6.2)	174 (10.8)	
Non-European [b]	60 (41.1)	261 (39.3)	471 (23.8)		47 (39.2)	222 (40.7)	365 (22.7)	
Missing	*1 (0.7)*	*5 (0.7)*	*6 (0.3)*		*0*	*3 (0.5)*	*6 (0.4)*	
Poverty [c]								
Yes	31 (24.6)	92 (15.2)	42 (2.2)	<0.001	24 (25.8)	70 (15.0)	30 (2.0)	<0.001
No	95 (75.4)	512 (84.8)	1860 (97.8)		69 (74.2)	398 (85.0)	1486 (98.0)	
Missing	*21 (14.3)*	*65 (9.7)*	*87 (4.4)*		*27 (22.5)*	*81 (14.8)*	*100 (6.2)*	
Household income in euros per month [d]								
Less than 3,000	100 (72.5)	296 (46.2)	258 (13.3)	<0.001	85 (75.2)	238 (46.9)	196 (12.6)	<0.001
3,000-5,999	37 (26.8)	329 (51.4)	1315 (67.6)		25 (22.1)	261 (51.5)	1051 (67.6)	
Equal or more than 6,000	1 (0.7)	15 (2.3)	371 (19.1)		3 (2.7)	8 (1.6)	307 (19.8)	
Missing	*9 (6.1)*	*29 (4.3)*	*45 (2.3)*		*7 (5.8)*	*42 (7.7)*	*62 (3.8)*	
Cohabitation status [e]								
Not cohabiting	39 (30.5)	142 (22.9)	144 (7.5)	<0.001	27 (28.7)	112 (23.2)	101 (6.6)	<0.001
Cohabiting	89 (69.5)	478 (77.1)	1771 (92.5)		67 (71.3)	370 (76.8)	1422 (93.4)	
Missing	*19 (12.9)*	*49 (7.3)*	*74 (3.7)*		*26 (21.7)*	*67 (12.2)*	*93 (5.8)*	
BMI, median [IQR], kg/m^2	26.0 [22.9, 30.1]	25.5 [22.6, 29.8]	22.9 [20.9, 25.3]	<0.001	25.8 [22.9, 30.9]	25.2 [22.5, 29.2]	22.9 [20.9, 25.2]	<0.001
Missing	*6 (4.1)*	*16 (2.4)*	*12 (0.6)*		*1 (0.8)*	*8 (1.5)*	*13 (0.8)*	

© 2025 Boxem AJ et al. *JAMA Network Open*.

	Participants, No. (%)							
	Women							
	Educational level							
	**Time to pregnancy N=2,805**				**Miscarriage N=2,285**			
	**Low**	**Middle**	**High**	**p-value**	**Low**	**Middle**	**High**	**p-value**
Characteristic	N=147	N=669	N=1,989		N=120	N=549	N=1,616	
Smoking								
No	47 (33.1)	282 (43.7)	1193 (61.2)	<0.001	35 (33.0)	216 (42.8)	960 (61.9)	<0.001
No, quit smoking before pregnancy	35 (24.6)	213 (33.0)	599 (30.7)		21 (19.8)	162 (32.1)	461 (29.7)	
Yes, smoked during pregnancy	60 (42.3)	150 (23.3)	156 (8.0)		50 (47.2)	127 (25.1)	131 (8.4)	
Missing	*5 (3.4)*	*24 (3.6)*	*41 (2.1)*		*14 (11.7)*	*44 (8.0)*	*64 (4.0)*	
Alcohol consumption								
No consumption < 3 months before pregnancy	57 (41.3)	207 (32.4)	316 (16.1)	<0.001	46 (43.8)	164 (32.5)	253 (16.2)	<0.001
Yes, consumption < 3 months before pregnancy	68 (49.3)	329 (51.5)	1364 (69.7)		44 (41.9)	242 (48.0)	1054 (67.5)	
Yes, consumption during pregnancy	13 (9.4)	103 (16.1)	278 (14.2)		15 (14.3)	98 (19.4)	255 (16.3)	
Missing	*9 (6.1)*	*30 (4.5)*	*31 (1.6)*		*15 (12.5)*	*45 (8.2)*	*54 (3.3)*	
Folic acid supplementation								
Never	8 (6.4)	11 (1.9)	7 (0.4)	<0.001	5 (5.2)	10 (2.1)	5 (0.3)	<0.001
Started prior to pregnancy	47 (37.6)	313 (52.9)	1389 (74.5)		31 (32.3)	246 (51.2)	1126 (74.2)	
Started in pregnancy	70 (56.0)	268 (45.3)	468 (25.1)		60 (62.5)	224 (46.7)	387 (25.5)	
Missing	*22 (15.0)*	*77 (11.5)*	*125 (6.3)*		*24 (20.0)*	*69 (12.6)*	*98 (6.1)*	
Parity								
Nulliparous	68 (50.7)	372 (58.3)	1411 (72.4)	<0.001	48 (49.5)	286 (56.3)	1131 (71.8)	<0.001
Multiparous	66 (49.3)	266 (41.7)	538 (27.6)		49 (50.5)	222 (43.7)	445 (28.2)	
Missing	*13 (8.8)*	*31 (4.6)*	*40 (2.0)*		*23 (19.2)*	*41 (7.5)*	*40 (2.5)*	
Miscarriage in previous pregnancy								

© 2025 Boxem AJ et al. *JAMA Network Open*.

	Participants, No. (%)							
	Women							
	Educational level							
	Time to pregnancy N=2,805				Miscarriage N=2,285			
	**Low**	**Middle**	**High**	**p-value**	**Low**	**Middle**	**High**	**p-value**
Characteristic	N=147	N=669	N=1,989		N=120	N=549	N=1,616	
No	98 (71.5)	482 (75.4)	1620 (83.0)	<0.001	74 (73.3)	381 (76.8)	1294 (83.3)	0.001
Yes	39 (28.5)	157 (24.6)	332 (17.0)		27 (26.7)	115 (23.2)	260 (16.7)	
Missing	*10 (6.8)*	*30 (4.5)*	*37 (1.9)*		*19 (15.8)*	*53 (9.7)*	*62 (3.8)*	
Time to pregnancy, median *(95% range)* [f]	5.8 [0.0, 86.5]	4.5 [0.0, 92.4]	3.1 [0.0, 53.4]	<0.001	5.2 [0.0, 98.0]	4.5 [0.0, 90.3]	3.0 [0.0, 49.7]	<0.001
0-12 months	84 (57.1)	394 (58.9)	1335 (67.1)		64 (68.8)	309 (65.3)	1146 (74.5)	<0.001
> 12 months	33 (22.4)	153 (22.9)	283 (14.2)		22 (23.7)	113 (23.9)	223 (14.5)	
ART leading to pregnancy	13 (8.8)	61 (9.1)	202 (10.2)		7 (7.5)	51 (10.8)	169 (11.0)	
Not pregnant	17 (11.6)	61 (9.1)	169 (8.5)		NA	NA	NA	
Overall subfertility [g]	63 (42.9)	270 (40.7)	624 (31.9)		29 (31.2)	164 (34.7)	392 (25.5)	
Missing	*0*	*0*	*0*		*27 (22.5)*	*76 (13.8)*	*78 (4.8)*	
Occurrence of miscarriage								
No miscarriage	121 (93.1)	561 (92.3)	1673 (91.9)	0.875	110 (91.7)	485 (88.3)	1436 (88.9)	0.576
Miscarriage	9 (6.9)	47 (7.7)	147 (8.1)		10 (8.3)	64 (11.7)	180 (11.1)	
Missing	*17 (11.6)*	*61 (9.1)*	*169 (8.5)*		*0*	*0*	*0*	
Timing of miscarriage, median [IQR], wk.	8.4 [7.6, 10.4]	8.6 [7.3, 10.0]	8.1 [7.0, 9.2]	0.353	8.3 [8.0, 8.9]	8.3 [6.6, 9.7]	8.1 [7.0, 9.1]	0.633
First trimester	6 (75.0)	42 (91.3)	139 (94.6)		7 (77.8)	58 (92.1)	171 (95.5)	0.068
Second trimester	2 (25.0)	4 (8.7)	8 (5.4)		2 (22.2)	5 (7.9)	8 (4.5)	
Missing	1 (11.1)	1 (2.1)	0 (0.0)		1 (10.0)	1 (1.6)	1 (0.6)	

Abbreviations: ART, assisted reproductive technology; BMI, body mass index (calculated as weight in kilograms divided by height in meters squared); NA, not applicable.

a. Included: European, German, Yugoslav, or Polish migration background.
b. Included: African; American, non-western; Asian, non-western; Chinese; Indonesian; American, western; Asian, western; Cape Verdean; Dutch Antilles; Moroccan; Oceanian; Surinamese, or Turkish migration background.
c. ‘Yes’ consists of ‘experiencing financial difficulties’ combined with ‘household income of less than 3000 euros per month’. ‘No’ consists of ‘experiencing financial difficulties’ combined with ‘household income of 3,000-5,999 euros per month’ and ‘equal or more than 6,000 euros per month’ as well as ‘not experiencing financial difficulties’ combined with ‘household income of ‘less than 3,000 euros per month’, ‘3,000-5,999 euros per month’ and ‘equal or more than 6,000 euros per month’.

© 2025 Boxem AJ et al. *JAMA Network Open*.

d. ‘Less than 3,000 euros per month’ consists of ‘less than 900 euros per month’, ‘1,000-1,999 euros per month’ and ‘2,000-2,999 euros per month’. ‘3,000-5,999 euros per month’ consists of ‘3,000-3,999 euros per month’, ‘4,000-4,999 euros per month’ and ‘5,000-5,999 euros per month’.
e. ‘Not cohabiting’ consisted of ‘no partner and/or not cohabiting’ and ‘married, living separately’. ‘Cohabiting’ consisted of ‘cohabiting, but not married’, ‘married and cohabiting with spouse’, and ‘registered partnership and cohabiting’.
f. Time to pregnancy in months was derived from pregnancy episodes with a natural conception.
g. For the low educational level: episodes with ART leading to pregnancy, episodes without pregnancy and use of ART (0 of 17 episodes), and episodes without pregnancy, without use of ART, and a duration of actively pursuing pregnancy of more than 12 months (17 of 17 episodes) were added to the subfertile group (time to pregnancy > 12 months and use of ART) in the analysis. For the middle educational level: episodes with ART leading to pregnancy, episodes without pregnancy and use of ART (0 of 61 episodes), and episodes without pregnancy, without use of ART and a duration of actively pursuing pregnancy of more than 12 months (56 of 61 episodes) were added to the subfertile group (time to pregnancy > 12 months and use of ART) in the analysis. For the high educational level: episodes with ART leading to pregnancy, episodes without pregnancy and use of ART (7 of 169 episodes), and episodes without pregnancy, without use of ART and a duration of actively pursuing pregnancy of more than 12 months (132 of 169 episodes) were added to the subfertile group (time to pregnancy > 12 months and use of ART) in the analysis.

© 2025 Boxem AJ et al. *JAMA Network Open*.

eTable 4. Population Characteristics Presented per Level of Education Level of Men

	Participants, No. (%)							
	Men							
	Educational level							
	Time to pregnancy N=2,633				Miscarriage N=2,151			
	Low	Middle	High	p-value	Low	Middle	High	p-value
Characteristic	N=234	N=794	N=1,605		N=192	N=629	N=1,130	
Age, median [IQR], y	33.2 [29.0, 37.9]	32.2 [28.8, 36.4]	33.7 [31.2, 36.7]	<0.001	32.9 [28.4, 37.6]	32.2 [28.7, 36.2]	33.7 [31.2, 36.7]	<0.001
Missing	*77 (32.9)*	*188 (23.7)*	*226 (14.1)*		*42 (21.9)*	*90 (14.3)*	*95 (8.4)*	
Migration background								
Dutch	103 (45.8)	402 (52.7)	1089 (69.7)	<0.001	81 (42.9)	323 (52.8)	922 (70.5)	<0.001
European [a]	17 (7.6)	48 (6.3)	151 (9.7)		15 (7.9)	29 (4.7)	120 (9.2)	
Non-European [b]	105 (46.7)	313 (41.0)	323 (20.7)		93 (49.2)	260 (42.5)	266 (20.3)	
Missing	*9 (3.8)*	*31 (3.9)*	*42 (2.6)*		*3 (1.6)*	*17 (2.7)*	*22 (1.7)*	
Poverty [c]								
Yes	33 (15.3) [h]	73 (10.1) [h]	18 (1.2) [h]	<0.001	28 (16.9) [h]	51 (9.3) [h]	14 (1.1) [h]	<0.001
No	182 (84.7) [h]	648 (89.9) [h]	1531 (98.8) [h]		138 (83.1) [h]	496 (90.7) [h]	1247 (98.9) [h]	
Missing	*19 (8.1)* [h]	*73 (9.2)* [h]	*56 (3.5)* [h]		*26 (13.5)* [h]	*82 (13.0)* [h]	*69 (5.2)* [h]	
Household income in euros per month [d]								
Less than 3,000	120 (53.1) [h]	261 (34.4) [h]	150 (9.5) [h]	<0.001	105 (58.0) [h]	194 (33.1) [h]	129 (10.0) [h]	<0.001
3,000-5,999	101 (44.7) [h]	461 (60.8) [h]	1080 (68.7) [h]		71 (39.2) [h]	368 (62.8) [h]	875 (67.9) [h]	
Equal or more than 6,000	5 (2.2) [h]	36 (4.7) [h]	342 (21.8) [h]		5 (2.8) [h]	24 (4.1) [h]	284 (22.0) [h]	
Missing	*8 (3.4)* [h]	*36 (4.5)* [h]	*33 (2.1)* [h]		*11 (5.7)* [h]	*43 (6.8)* [h]	*42 (3.2)* [h]	
Cohabitation status [e]								
Not cohabiting	48 (21.8) [h]	108 (14.6) [h]	60 (3.9) [h]	<0.001	41 (24.0) [h]	79 (14.1) [h]	49 (3.9) [h]	<0.001
Cohabiting	172 (78.2) [h]	632 (85.4) [h]	1497 (96.1) [h]		130 (76.0) [h]	482 (85.9) [h]	1215 (96.1) [h]	
Missing	*14 (6.0)* [h]	*54 (6.8)* [h]	*48 (3.0)* [h]		*21 (10.9)* [h]	*68 (10.8)* [h]	*66 (5.0)* [h]	
BMI, median [IQR], kg/m^2	26.1 [24.3, 28.7]	26.3 [23.8, 29.2] [h]	24.4 [22.7, 26.4] [h]	<0.001	26.0 [24.1, 28.7] [h]	26.3 [23.7, 29.3] [h]	24.4 [22.7, 26.4] [h]	<0.001

© 2025 Boxem AJ et al. *JAMA Network Open*.

	Participants, No. (%)							
	Men							
	Educational level							
	Time to pregnancy N=2,633				Miscarriage N=2,151			
	Low	Middle	High	p-value	Low	Middle	High	p-value
Characteristic	N=234	N=794	N=1,605		N=192	N=629	N=1,130	
Missing	*85 (36.3)*	*220 (27.7)* [h]	*274 (17.1)* [h]		*48 (25.0)* [h]	*125 (19.9)* [h]	*146 (12.9)* [h]	
Smoking								
No	70 (34.3)	282 (40.8)	924 (60.5)	<0.001	50 (29.2)	217 (39.2)	734 (58.2)	<0.001
No, quit smoking before pregnancy	36 (17.6)	161 (23.3)	356 (23.3)		33 (19.3)	137 (24.7)	313 (24.8)	
Yes, smoked during pregnancy	98 (48.0)	248 (35.9)	247 (16.2)		88 (51.5)	200 (36.1)	215 (17.0)	
Missing	*30 (12.8)*	*103 (13.0)*	*78 (4.9)*		*21 (10.9)*	*75 (11.9)*	*68 (5.1)*	
Alcohol consumption								
No consumption < 3 months before pregnancy	66 (29.3)	135 (18.0)	129 (8.2)	<0.001	48 (27.6)	96 (16.9)	107 (8.4)	<0.001
Yes, consumption < 3 months before pregnancy	159 (70.7)	615 (82.0)	1444 (91.8)		126 (72.4)	472 (83.1)	1172 (91.6)	
Yes, consumption during pregnancy	NA	NA	NA		NA	NA	NA	
Missing	*9 (3.8)*	*44 (5.5)*	*32 (2.0)*		*18 (9.4)*	*61 (9.7)*	*51 (3.8)*	
Folic acid supplementation								
Never	9 (4.3) [h]	9 (1.3) [h]	4 (0.3) [h]	<0.001	9 (5.4) [h]	6 (1.1) [h]	3 (0.2) [h]	<0.001
Started prior to pregnancy	103 (49.5) [h]	393 (55.4) [h]	1178 (76.6) [h]		74 (44.0) [h]	306 (54.8) [h]	969 (76.6) [h]	
Started in pregnancy	96 (46.2) [h]	308 (43.4) [h]	355 (23.1) [h]		85 (50.6) [h]	246 (44.1) [h]	293 (23.2) [h]	
Missing	*26 (11.1)* [h]	*84 (10.6)* [h]	*68 (4.2)* [h]		*24 (12.5)* [h]	*71 (11.3)* [h]	*65 (4.9)* [h]	
Parity								
Nulliparous	118 (52.9) [h]	472 (62.2) [h]	1157 (73.0) [h]	<0.001	95 (54.0) [h]	352 (60.3) [h]	946 (72.5) [h]	<0.001
Multiparous	105 (47.1) [h]	287 (37.8) [h]	427 (27.0) [h]		81 (46.0) [h]	232 (39.7) [h]	358 (27.5) [h]	
Missing	*11 (4.7)* [h]	*35 (4.4)* [h]	*21 (1.3)* [h]		*16 (8.3)* [h]	*45 (7.2)* [h]	*26 (2.0)* [h]	

© 2025 Boxem AJ et al. *JAMA Network Open*.

	Participants, No. (%)							
	Men							
	Educational level							
	**Time to pregnancy N=2,633**				**Miscarriage N=2,151**			
	**Low**	**Middle**	**High**	**p-value**	**Low**	**Middle**	**High**	**p-value**
Characteristic	N=234	N=794	N=1,605		N=192	N=629	N=1,130	
Miscarriage in previous pregnancy								
No	164 (72.2)[h]	584 (76.8)[h]	1330 (83.9)[h]	<0.001	137 (77.8)[h]	450 (78.0)[h]	1074 (83.4)[h]	0.009
Yes	63 (27.8)[h]	176 (23.2)[h]	255 (16.1)[h]		39 (22.2)[h]	127 (22.0)[h]	213 (16.6)[h]	
Missing	*7 (3.0)*[h]	*34 (4.3)*[h]	*20 (1.2)*[h]		*16 (8.3)*[h]	*52 (8.3)*[h]	*43 (3.2)*[h]	
Time to pregnancy, median *(95% range)*[f]	4.8 [0.0, 94.4][h]	4.3 [0.0, 84.8][h]	2.9 [0.0, 47.3][h]		3.8 [0.0, 116.5][h]	4.2 [0.0, 77.2][h]	2.9 [0.0, 46.6][h]	<0.001
0-12 months	142 (60.7)[h]	497 (62.6)[h]	1122 (69.9)[h]	<0.001	119 (72.6)[h]	395 (69.1)[h]	965 (75.9)[h]	
> 12 months	41 (17.5)[h]	167 (21.0)[h]	221 (13.8)[h]		26 (15.9)[h]	132 (23.1)[h]	175 (13.8)[h]	
ART leading to pregnancy	27 (11.5)[h]	57 (7.2)[h]	153 (9.5)[h]		19 (11.6)[h]	45 (7.9)[h]	131 (10.3)[h]	
Not pregnant	24 (10.3)[h]	73 (9.2)[h]	109 (6.8)[h]		NA[h]	NA[h]	NA[h]	
Overall subfertility[g]	90 (38.8)[h]	288 (36.7)[h]	460 (29.1)[h]		45 (27.4)[h]	177 (30.9)[h]	306 (24.1)[h]	
Missing	*0*[h]	*0*[h]	*0*[h]		*28 14.6*[h]	*57 9.1*[h]	*59 4.4*[h]	
Occurrence of miscarriage								
No miscarriage	194 (92.4)[h]	668 (92.6)[h]	1381 (92.3)[h]		173 (90.1)[h]	562 (89.3)[h]	1188 (89.3)[h]	0.946
Miscarriage	16 (7.6)[h]	53 (7.4)[h]	115 (7.7)[h]		19 (9.9)[h]	67 (10.7)[h]	142 (10.7)[h]	
Missing	*24 (10.3*[h]	*73 (9.2)*[h]	*109 (6.8)*[h]		*0*[h]	*0*[h]	*0*[h]	
Timing of miscarriage, median [IQR], wk.	7.7 [6.7, 8.9][h]	8.5 [7.4, 9.9][h]	8.1 [7.0, 9.1][h]	0.185	7.7 [6.9, 9.1][h]	8.4 [7.3, 9.9][h]	8.1 [6.9, 9.1][h]	0.234
First trimester	14 (87.5)[h]	48 (92.3)[h]	108 (94.7)[h]		17 (89.5)[h]	61 (92.4)[h]	135 (95.7)[h]	0.409
Second trimester	2 (12.5)[h]	4 (7.7)[h]	6 (5.3)[h]		2 (10.5)[h]	5 (7.6)[h]	6 (4.3)[h]	
Missing	*0 (0.0)*[h]	*1 (1.9)*[h]	*1 (0.9)*[h]		*0 (0.0)*[h]	*1 (1.5)*[h]	*1 (0.7)*[h]	

Abbreviations: ART, assisted reproductive technology; BMI, body mass index (calculated as weight in kilograms divided by height in meters squared); NA, not applicable.

a. Included: European, German, Yugoslav, or Polish migration background.
b. Included: African; American, non-western; Asian, non-western; Chinese; Indonesian; American, western; Asian, western; Cape Verdean; Dutch Antilles; Moroccan; Oceanian; Surinamese, or Turkish migration background.

© 2025 Boxem AJ et al. *JAMA Network Open*.

c. ‘Yes’ consists of ‘experiencing financial difficulties’ combined with ‘household income of less than 3000 euros per month’. ‘No’ consists of ‘experiencing financial difficulties’ combined with ‘household income of 3,000-5,999 euros per month’ and ‘equal or more than 6,000 euros per month’ as well as ‘not experiencing financial difficulties’ combined with ‘household income of ‘less than 3,000 euros per month’, ‘3,000-5,999 euros per month’ and ‘equal or more than 6,000 euros per month’.
d. ‘Less than 3,000 euros per month’ consists of ‘less than 900 euros per month’, ‘1,000-1,999 euros per month’ and ‘2,000-2,999 euros per month’. ‘3,000-5,999 euros per month’ consists of ‘3,000-3,999 euros per month’, ‘4,000-4,999 euros per month’ and ‘5,000-5,999 euros per month’.
e. ‘Not cohabiting’ consisted of ‘no partner and/or not cohabiting’ and ‘married, living separately’. ‘Cohabiting’ consisted of ‘cohabiting, but not married’, ‘married and cohabiting with spouse’, and ‘registered partnership and cohabiting’.
f. Time to pregnancy in months was derived from pregnancy episodes with a natural conception.
g. For the low educational level: episodes with ART leading to pregnancy, episodes without pregnancy and use of ART (1 of 24 episodes), and episodes without pregnancy, without use of ART, and a duration of actively pursuing pregnancy of more than 12 months (21 of 24 episodes) were added to the subfertile group (time to pregnancy > 12 months and use of ART) in the analysis. For the middle educational level: episodes with ART leading to pregnancy, episodes without pregnancy and use of ART (6 of 73 episodes), and episodes without pregnancy, without use of ART and a duration of actively pursuing pregnancy of more than 12 months (63 of 73 episodes) were added to the subfertile group (time to pregnancy > 12 months and use of ART) in the analysis. For the high educational level: episodes with ART leading to pregnancy, episodes without pregnancy and use of ART (4 of 109 episodes), and episodes without pregnancy, without use of ART and a duration of actively pursuing pregnancy of more than 12 months (82 of 109 episodes) were added to the subfertile group (time to pregnancy > 12 months and use of ART) in the analysis.
h. Household income, poverty, cohabitation status, folic acid supplementation, parity, miscarriage in previous pregnancy, time to pregnancy in months, occurrence of miscarriage, timing of miscarriage in weeks of men were derived from their partner.

© 2025 Boxem AJ et al. *JAMA Network Open*.

eTable 5. Population Characteristics Presented per Level of Household Income

	Episodes, No. (%)							
	Household income							
	Time to pregnancy N=2,752				Miscarriage N=2,200			
	**<3,000**	**3000-5,999**	**≥6,000**	**p-value**	**<3,000**	**3000-5,999**	**≥6,000**	**p-value**
Characteristic	N=664	N=1,697	N=391		N=527	N=1,351	N=322	
Age, median [IQR], y	31.2 [27.6, 35.7]	31.2 [29.3, 33.8]	32.6 [30.8, 34.9]	<0.001	30.8 [27.1, 35.0]	31.1 [29.2, 33.6]	32.7 [30.8, 34.8]	<0.001
Missing	*0*	*0*	*0*		*0*	*0*	*0*	
Migration background								
Dutch	276 (41.9)	1140 (67.4)	270 (69.2)	<0.001	229 (43.8)	926 (68.7)	229 (71.3)	<0.001
European [a]	61 (9.3)	169 (10.0)	50 (12.8)		47 (9.0)	127 (9.4)	37 (11.5)	
Non-European [b]	322 (48.9)	383 (22.6)	70 (17.9)		247 (47.2)	294 (21.8)	55 (17.1)	
Missing	*5 (0.8)*	*5 (0.3)*	*1 (0.3)*		*4 (0.8)*	*4 (0.3)*	*1 (0.3)*	
Poverty [c]								
Yes	169 (27.2)	0 (0.0)	0 (0.0)	<0.001	127 (27.2)	0 (0.0)	0 (0.0)	<0.001
No	452 (72.8)	1662 (100.0)	379 (100.0)		340 (72.8)	1326 (100.0)	310 (100.0)	
Missing	*43 (6.5)*	*35 (2.1)*	*12 (3.1)*		*60 (11.4)*	*25 (1.9)*	*12 (3.7)*	
Educational level [d]								
Low	100 (15.3)	37 (2.2)	1 (0.3)	<0.001	85 (16.4)	25 (1.9)	3 (0.9)	<0.001
Middle	296 (45.3)	329 (19.6)	15 (3.9)		238 (45.9)	261 (19.5)	8 (2.5)	
High	258 (39.4)	1315 (78.2)	371 (95.9)		196 (37.8)	1051 (78.6)	307 (96.5)	
Missing	*10 (1.5)*	*16 (0.9)*	*4 (1.0)*		*8 (1.5)*	*14 (1.0)*	*4 (1.2)*	
Cohabitation status [e]								
Not cohabiting	266 (44.6)	46 (2.8)	7 (1.9)	<0.001	196 (43.7)	31 (2.4)	7 (2.3)	<0.001
Cohabiting	331 (55.4)	1598 (97.2)	368 (98.1)		253 (56.3)	1280 (97.6)	300 (97.7)	
Missing	*67 (10.1)*	*53 (3.1)*	*16 (4.1)*		*78 (14.8)*	*40 (3.0)*	*15 (4.7)*	
BMI, median [IQR], kg/m^2	25.3 [21.8, 29.7]	23.3 [21.2, 26.1]	22.1 [20.5, 24.1]	<0.001	24.9 [21.6, 29.5]	23.3 [21.2, 25.9]	22.2 [20.7, 24.1]	<0.001
Missing	*13 (2.0)*	*12 (0.7)*	*6 (1.5)*		*6 (1.1)*	*5 (0.4)*	*1 (0.3)*	
Smoking								
No	290 (45.5)	965 (57.7)	247 (65.5)	<0.001	206 (42.6)	777 (58.2)	209 (67.4)	<0.001

© 2025 Boxem AJ et al. *JAMA Network Open*.

	Episodes, No. (%)							
	Household income							
	Time to pregnancy N=2,752				Miscarriage N=2,200			
	**<3,000**	**3000-5,999**	**≥6,000**	**p-value**	**<3,000**	**3000-5,999**	**≥6,000**	**p-value**
Characteristic	N=664	N=1,697	N=391		N=527	N=1,351	N=322	
No, quit smoking before pregnancy	192 (30.1)	527 (31.5)	105 (27.9)		136 (28.1)	412 (30.9)	79 (25.5)	
Yes, smoked during pregnancy	155 (24.3)	180 (10.8)	25 (6.6)		142 (29.3)	145 (10.9)	22 (7.1)	
Missing	*27 (4.1)*	*25 (1.5)*	*14 (3.6)*		*43 (8.2)*	*17 (1.3)*	*12 (3.7)*	
Alcohol consumption								
No consumption < 3 months before pregnancy	221 (35.0)	307 (18.4)	36 (9.4)	<0.001	181 (37.2)	239 (17.9)	31 (9.9)	<0.001
Yes, consumption < 3 months before pregnancy	339 (53.7)	1113 (66.6)	285 (74.6)		233 (47.9)	866 (64.8)	227 (72.3)	
Yes, consumption during pregnancy	71 (11.3)	252 (15.1)	61 (16.0)		72 (14.8)	231 (17.3)	56 (17.8)	
Missing	*33 (5.0)*	*25 (1.5)*	*9 (2.3)*		*41 (7.8)*	*15 (1.1)*	*8 (2.5)*	
Folic acid supplementation								
Never	20 (3.6)	6 (0.4)	0 (0.0)	<0.001	15 (3.3)	4 (0.3)	1 (0.3)	<0.001
Started prior to pregnancy	276 (49.1)	1142 (71.2)	303 (82.6)		207 (45.7)	921 (70.7)	254 (83.0)	
Started in pregnancy	266 (47.3)	457 (28.5)	64 (17.4)		231 (51.0)	378 (29.0)	51 (16.7)	
Missing	*102 (15.4)*	*92 (5.4)*	*24 (6.1)*		*74 (14.0)*	*48 (3.6)*	*16 (5.0)*	
Parity								
Nulliparous	373 (59.6)	1164 (69.7)	282 (74.6)	<0.001	276 (58.7)	927 (69.5)	226 (72.9)	<0.001
Multiparous	253 (40.4)	506 (30.3)	96 (25.4)		194 (41.3)	406 (30.5)	84 (27.1)	
Missing	*38 (5.7)*	*27 (1.6)*	*13 (3.3)*		*57 (10.8)*	*18 (1.3)*	*12 (3.7)*	
Miscarriage in previous pregnancy								
No	471 (75.1)	1380 (82.5)	315 (83.1)	<0.001	367 (77.8)	1104 (82.8)	253 (81.6)	0.055
Yes	156 (24.9)	293 (17.5)	64 (16.9)		105 (22.2)	230 (17.2)	57 (18.4)	
Missing	*37 (5.6)*	*24 (1.4)*	*12 (3.1)*		*55 (10.4)*	*17 (1.3)*	*12 (3.7)*	

© 2025 Boxem AJ et al. *JAMA Network Open*.

	Episodes, No. (%)							
	Household income							
	Time to pregnancy<br>N=2,752				Miscarriage<br>N=2,200			
	<3,000	3000-5,999	≥6,000	p-value	<3,000	3000-5,999	≥6,000	p-value
Characteristic	N=664	N=1,697	N=391		N=527	N=1,351	N=322	
Time to pregnancy, median *(95% range)* [f]	4.8 [0.0, 106.6]	3.0 [0.0, 50.9]	3.5 [0.0, 59.0]		4.3 [0.0, 114.2]	3.0 [0.0, 47.6]	3.5 [0.0, 55.4]	
0-12 months	357 (53.8)	1161 (68.4)	266 (68.0)	<0.001	286 (64.4)	984 (74.7)	230 (74.2)	<0.001
> 12 months	149 (22.4)	246 (14.5)	60 (15.3)		106 (23.9)	194 (14.7)	49 (15.8)	
ART leading to pregnancy	65 (9.8)	166 (9.8)	38 (9.7)		52 (11.7)	140 (10.6)	31 (10.0)	
Not pregnant	93 (14.0)	124 (7.3)	27 (6.9)		NA	NA	NA	
Overall subfertility [g]	301 (45.7)	516 (30.8)	116 (30.4)		158 (35.6)	334 (25.3)	80 (25.8)	
Missing	*0*	*0*	*0*		*83 (15.7)*	*33 (2.4)*	*12 (3.7)*	
Occurrence of miscarriage								
No miscarriage	525 (91.9)	1445 (91.9)	338 (92.9)	0.817	478 (90.7)	1217 (90.1)	294 (91.3)	0.772
Miscarriage	46 (8.1)	128 (8.1)	26 (7.1)		49 (9.3)	134 (9.9)	28 (8.7)	
Missing	*93 (14.0)*	*124 (7.3)*	*27 (6.9)*		*0*	*0*	*0*	
Timing of miscarriage, median [IQR], wk.	8.1 [6.7, 9.9]	8.3 [7.1, 9.6]	8.1 [7.3, 9.0]	0.940	8.3 [6.7, 9.9]	8.1 [7.0, 9.6]	8.1 [7.1, 9.1]	0.992
First trimester	38 (86.4)	121 (94.5)	25 (96.2)	0.150	41 (87.2)	127 (94.8)	27 (96.4)	0.159
Second trimester	6 (13.6)	7 (5.5)	1 (3.8)		6 (12.8)	7 (5.2)	1 (3.6)	
Missing	*2 (4.3)*	*0 (0.0)*	*0 (0.0)*		*2 (4.1)*	*0 (0.0)*	*0 (0.0)*	

Abbreviations: ART, assisted reproductive technology; BMI, body mass index (calculated as weight in kilograms divided by height in meters squared); NA, not applicable.

a. Included: European, German, Yugoslav, or Polish migration background.
b. Included: African; American, non-western; Asian, non-western; Chinese; Indonesian; American, western; Asian, western; Cape Verdean; Dutch Antilles; Moroccan; Oceanian; Surinamese, or Turkish migration background.
c. 'Yes' consists of 'experiencing financial difficulties' combined with 'household income of less than 3000 euros per month'. 'No' consists of 'experiencing financial difficulties' combined with 'household income of 3,000-5,999 euros per month' and 'equal or more than 6,000 euros per month' as well as 'not experiencing financial difficulties' combined with 'household income of 'less than 3,000 euros per month', '3,000-5,999 euros per month' and 'equal or more than 6,000 euros per month'.
d. 'Low' consists of 'no primary education finished', 'primary education finished' and 'secondary education phase 1 finished', 'middle' consists of 'secondary education phase 2 finished' and 'high' consists of 'higher education phase 1 finished' and 'higher education phase 2 finished'.
e. 'Not cohabiting' consisted of 'no partner and/or not cohabiting' and 'married, living separately'. 'Cohabiting' consisted of 'cohabiting, but not married', 'married and cohabiting with spouse', and 'registered partnership and cohabiting'.
f. Time to pregnancy in months was derived from pregnancy episodes with a natural conception.
g. For the income of less than 3000 euros per month: episodes with ART leading to pregnancy, episodes without pregnancy and use of ART (1 of 93 episodes), and episodes without pregnancy, without use of ART, and a duration of actively pursuing pregnancy of more than 12 months (86 of 93 episodes) were added to the subfertile group (time to pregnancy > 12 months and use of ART) in the

© 2025 Boxem AJ et al. *JAMA Network Open*.

analysis. For the income of 3000-5999 euros per month: episodes with ART leading to pregnancy, episodes without pregnancy and use of ART (5 of 124 episodes), and episodes without pregnancy, without use of ART and a duration of actively pursuing pregnancy of more than 12 months (99 of 124 episodes) were added to the subfertile group (time to pregnancy > 12 months and use of ART) in the analysis. For the income of equal or more than 6000 euros per month: episodes with ART leading to pregnancy, episodes without pregnancy and use of ART (1 of 27 episodes), and episodes without pregnancy, without use of ART and a duration of actively pursuing pregnancy of more than 12 months (17 of 27 episodes) were added to the subfertile group (time to pregnancy > 12 months and use of ART) in the analysis.

© 2025 Boxem AJ et al. *JAMA Network Open*.

eTable 6. Nonresponse Analysis of Participants Included and Excluded From the Study Populations

	Women			Men		
	Participants, No. %			Participants, No. %		
	Non-responders	Responders	p-value	Non-responders	Responders	p-value
Characteristic	N=163	N=3,018		N=161	N=3,000	
Age, median [IQR], y	29.7 [27.1, 32.7]	31.4 [29.0, 34.3]	<0.001	31.3 [24.4, 34.3]	33.3 [30.3, 36.7]	0.006
Missing	*0*	*0*		*129 (80.1)*	*704 (23.5)*	
Migration background						
Dutch	69 (42.3)	1790 (59.6)	<0.001	59 (45.4)	1678 (60.7)	0.001
European [a]	17 (10.4)	312 (10.4)		12 (9.2)	237 (8.6)	
Non-European [b]	77 (47.2)	901 (30.0)		59 (45.4)	851 (30.8)	
Missing	*0*	*15 (0.5)*		*31 (19.3)*	*234 (7.8)*	
Poverty [c]						
Yes	13 (11.7)	187 (6.8)	0.074	13 (11.7)[h]	186 (6.8) [h]	0.074
No	98 (88.3)	2555 (93.2)		98 (88.3) [h]	2538 (93.2) [h]	
Missing	*52 (31.9)*	*276 (9.1)*		*50 (31.1)* [h]	*276 (9.2)* [h]	
Educational level [d]						
Low	13 (8.1)	174 (5.8)	0.347	14 (10.2)	267 (9.5)	0.696
Middle	44 (27.3)	745 (24.9)		46 (33.6)	860 (30.6)	
High	104 (64.6)	2067 (69.2)		77 (56.2)	1680 (59.9)	
Missing	*2 (1.2)*	*32 (1.1)*		*24 (14.9)*	*193 (6.4)*	
Household income, euros per month [e]						
Less than 3,000	64 (42.7)	747 (25.9)	<0.001	64 (42.7) [h]	741 (25.9) [h]	<0.001
3,000-5,999	72 (48.0)	1730 (60.1)		72 (48.0) [h]	1716 (60.0) [h]	
Equal or more than 6,000	14 (9.3)	403 (14.0)		14 (9.3) [h]	402 (14.1) [h]	
Missing	*13 (8.0)*	*138 (4.6)*		*11 (6.8)* [h]	*141 (4.7)* [h]	
Cohabitation status [f]						
Not cohabiting	31 (27.2)	364 (13.1)	<0.001	30 (26.5) [h]	358 (13.0) [h]	<0.001
Cohabiting	83 (72.8)	2413 (86.9)		83 (73.5) [h]	2394 (87.0) [h]	
Missing	*49 (30.1)*	*241 (8.0)*		*48 (29.8)* [h]	*248 (8.3)* [h]	
BMI, median [IQR], kg/m^2	23.8 [21.8, 27.5]	23.5 [21.2, 26.7]	0.154	26.1 [23.1, 29.3]	25.0 [23.0, 27.5]	0.380

© 2025 Boxem AJ et al. *JAMA Network Open*.

	Women			Men		
	Participants, No. %			Participants, No. %		
	Non-responders	Responders	p-value	Non-responders	Responders	p-value
Characteristic	N=163	N=3,018		N=161	N=3,000	
Missing	*33 (20.2)*	*51 (1.7)*		*139 (86.3)*	*821 (27.4)*	
Smoking						
No	65 (52.0)	1584 (55.3)	0.563	48 (82.8)	1328 (52.2)	<0.001
No, quit smoking before pregnancy	44 (35.2)	879 (30.7)		2 (3.4)	580 (22.8)	
Yes, smoked during pregnancy	16 (12.8)	401 (14.0)		8 (13.8)	637 (25.0)	
Missing	*38 (23.2)*	*154 (5.1)*		*103 (64.0)*	*455 (15.2)*	
Alcohol consumption						
No consumption < 3 months before pregnancy	27 (21.8)	634 (22.1)	0.016	11 (11.2)	361 (13.4)	0.629
Yes, consumption < 3 months before pregnancy	90 (72.6)	1814 (63.3)		87 (88.8)	2324 (86.6)	
Yes, consumption during pregnancy	7 (5.6)	416 (14.5)		NA	NA	
Missing	*39 (23.9)*	*154 (5.1)*		*63 (39.1)*	*315 (10.5)*	
Folic acid supplementation						
Never	0 (0.0)	28 (1.0)	0.020	0 (0.0) [h]	28 (1.1) [h]	0.022
Started prior to pregnancy	16 (45.7)	1790 (66.7)		16 (45.7) [h]	1770 (66.5) [h]	
Started in pregnancy	19 (54.3)	867 (32.3)		19 (54.3) [h]	864 (32.5) [h]	
Missing	*128 (78.5)*	*333 (11.0)*		*126 (78.3)* [h]	*11.3 (338)* [h]	
Parity						
Nulliparous	99 (82.5)	1947 (67.7)	0.001	98 (82.4) [h]	1924 (67.7) [h]	0.001
Multiparous	21 (17.5)	928 (32.3)		21 (17.6) [h]	918 (32.3) [h]	
Missing	*43 (26.4)*	*143 (4.7)*		*42 (26.1)* [h]	*158 (5.3)* [h]	
Miscarriage in previous pregnancy						
No	102 (87.9)	2303 (80.9)	0.075	101 (87.8) [h]	2276 (80.7) [h]	0.074
Yes	14 (12.1)	544 (19.1)		14 (12.2) [h]	544 (19.3) [h]	
Missing	*47 (28.8)*	*171 (5.7)*		*46 (28.6)* [h]	*180 (6.0)* [h]	
Time to pregnancy, median (95% range) [g]	NA	3.5 [0.0, 67.3]		NA [h]	3.5 [0.0, 66.9] [h]	
0-12 months	0	1837 (64.8)	NA	0 [h]	1830 (65.2) [h]	<0.001
> 12 months	0	473 (16.7)		0 [h]	469 (16.7) [h]	
ART leading to pregnancy	0	277 (9.8)		0 [h]	264 (9.4) [h]	

© 2025 Boxem AJ et al. *JAMA Network Open*.

	Women			Men		
	Participants, No. %			Participants, No. %		
	Non-responders	Responders	p-value	Non-responders	Responders	p-value
Characteristic	N=163	N=3,018		N=161	N=3,000	
Not pregnant	101 (100.0)	248 (8.7)		100 (100.0) [h]	245 (8.7) [h]	
Overall subfertility	0	963 (34.4)		0 [h]	960 (34.4) [h]	
Missing	*62 (38.0)*	*183 (6.1)*		*61 (37.9)* [h]	*192 (6.4)* [h]	
Occurrence of miscarriage						
No miscarriage	62 (100.0)	2516 (90.8)	0.023	61 (100.0) [h]	2494 (91.2) [h]	0.029
Miscarriage	0	254 (9.2)		0 (0.0) [h]	240 (8.8) [h]	
Missing	*101 (62.0)*	*248 (8.2)*		*100 (62.1)* [h]	*266 (8.9)* [h]	
Timing of miscarriage, median [IQR], wk.	NA	8.1 [7.0, 9.4]	NA	0 [h]	8.1 [7.0, 9.4] [h]	NA
First trimester	0	236 (94.0)		0 [h]	224 (94.1) [h]	
Second trimester	0	15 (6.0)		0 [h]	14 (5.9) [h]	
Missing	*0*	*3 (1.2)*		*0* [h]	*2 (0.8)* [h]	

Abbreviations: ART, assisted reproductive technology; BMI, body mass index (calculated as weight in kilograms divided by height in meters squared); NA, not applicable.
Women were included in preconception and pregnancy between 2017 and 2021.

a. Included: European, German, Yugoslav, or Polish migration background.
b. Included: African; American, non-western; Asian, non-western; Chinese; Indonesian; American, western; Asian, western; Cape Verdean; Dutch Antilles; Moroccan; Oceanian; Surinamese, or Turkish migration background.
c. ‘Yes’ consists of ‘experiencing financial difficulties’ combined with ‘household income of less than 3000 euros per month’. ‘No’ consists of ‘experiencing financial difficulties’ combined with ‘household income of 3,000-5,999 euros per month’ and ‘equal or more than 6,000 euros per month’ as well as ‘not experiencing financial difficulties’ combined with ‘household income of ‘less than 3,000 euros per month’, ‘3,000-5,999 euros per month’ and ‘equal or more than 6,000 euros per month’.
d. ‘Low’ consists of ‘no primary education finished’, ‘primary education finished’ and ‘secondary education phase 1 finished’, ‘middle’ consists of ‘secondary education phase 2 finished’ and ‘high’ consists of ‘higher education phase 1 finished’ and ‘higher education phase 2 finished’.
e. ‘Less than 3,000 euros per month’ consists of ‘less than 900 euros per month’, ‘1,000-1,999 euros per month’ and ‘2,000-2,999 euros per month’. ‘3,000-5,999 euros per month’ consists of ‘3,000-3,999 euros per month’, ‘4,000-4,999 euros per month’ and ‘5,000-5,999 euros per month’.
f. ‘Not cohabiting’ consisted of ‘no partner and/or not cohabiting’ and ‘married, living separately’. ‘Cohabiting’ consisted of ‘cohabiting, but not married’, ‘married and cohabiting with spouse’, and ‘registered partnership and cohabiting’.
g. Time to pregnancy in months was derived from pregnancy episodes with a natural conception.
h. Household income, poverty, cohabitation status, folic acid supplementation, parity, miscarriage in previous pregnancy, time to pregnancy in months, occurrence of miscarriage, timing of miscarriage of weeks in men were derived from their partner.

© 2025 Boxem AJ et al. *JAMA Network Open*.

eTable 7. Associations of Poverty, Educational Level of Women and Men, and Household Income With Fecundability Ratios

	Total, n	Not conceived, n (%)	Basic model FR (95% CI)	Confounder model FR (95% CI)	Demographic factors model FR (95% CI)	Lifestyle factors model FR (95% CI)	Fully adjusted model FR (95% CI)
**Poverty**	2,401	222 (9.2)					
Yes	158	18 (11.4)	0.61 (0.51-0.72)	0.61 (0.51-0.72)	0.75 (0.63-0.90)	0.67 (0.56-0.80)	0.80 (0.66-0.96)
No	2,243	204 (9.1)	Reference	Reference	Reference	Reference	Reference
**Educational level of women**	2,522	240 (9.5)					
Low	134	17 (12.7)	0.68 (0.57-0.82)	0.61 (0.50-0.74)	0.72 (0.59-0.87)	0.68 (0.56-0.83)	0.78 (0.64-0.96)
Middle	608	61 (10.0)	0.78 (0.71-0.86)	0.68 (0.61-0.75)	0.75 (0.68-0.84)	0.75 (0.67-0.84)	0.81 (0.72-0.90)
High	1,780	162 (9.1)	Reference	Reference	Reference	Reference	Reference
**Educational level of men**	2,390	200 (8.4)					
Low	206	23 (11.2)	0.73 (0.62-0.85)	0.72 (0.62-0.85)	0.79 (0.67-0.93)	0.71 (0.60-0.83)	0.78 (0.66-0.92)
Middle	736	72 (9.8)	0.75 (0.68-0.82)	0.72 (0.66-0.80)	0.76 (0.69-0.84)	0.72 (0.65-0.79)	0.75 (0.68-0.83)
High	1,448	105 (7.3)	Reference	Reference	Reference	Reference	Reference
**Educational level of women and men**	2,390	200 (8.4)					
Both without high	547	54 (9.9)	0.69 (0.62-0.77)	0.60 (0.54-0.67)	0.66 (0.58-0.74)	0.64 (0.56-0.73)	0.69 (0.61-0.79)
Only women with high	395	41 (10.4)	0.80 (0.71-0.90)	0.80 (0.71-0.90)	0.84 (0.74-0.94)	0.81 (0.71-0.91)	0.83 (0.73-0.94)
Only men with high	131	8 (6.1)	0.85 (0.70-1.02)	0.81 (0.67-0.97)	0.85 (0.71-1.03)	0.86 (0.71-1.04)	0.89 (0.73-1.08)
Both with high	1317	97 (7.4)	Reference	Reference	Reference	Reference	Reference
**Household income in euros per month**	2,476	237 (9.6)					
Less than 3,000	598	92 (15.4)	0.65 (0.57-0.75)	0.59 (0.51-0.68)	0.72 (0.61-0.84)	0.65 (0.56-0.76)	0.78 (0.66-0.92)
3,000-5,999	1526	119 (7.8)	1.02 (0.90-1.15)	0.95 (0.84-1.07)	0.95 (0.84-1.07)	1.00 (0.88-1.13)	0.99 (0.88-1.13)
Equal or more than 6,000	352	26 (7.4)	Reference	Reference	Reference	Reference	Reference

Poverty:

- The confounder model was adjusted for participants' age and parity. Parity was not significant.
- The demographic model was adjusted for participants' age, migration background, and cohabitation status. Cohabitation status showed a possible violation (p=0.027) but the global test was not significant (0.247), so we decided to keep it in the model and assumed approximate proportionality.
- The lifestyle model was adjusted for participants' age, body mass index, alcohol consumption, smoking, and folic acid supplementation. Folic acid supplementation showed a possible violation (p=0.043) but the global test was not significant (0.131), so we decided to keep it in the model and assumed approximate proportionality.
- The fully adjusted model was adjusted for participants' age, migration background, cohabitation status, body mass index, alcohol consumption, smoking, and folic acid supplementation. The global test was significant showing non-proportionality (p=0.041), so cohabitation status was included as a stratification factor in the final model. After stratification, the global test was not significant (p=0.152).

© 2025 Boxem AJ et al. *JAMA Network Open*.

Educational level of women:

- The confounder model was adjusted for participants' age and parity. Parity was not significant.
- The demographic model was adjusted for participants' age, migration background, and cohabitation status. Cohabitation status showed a possible violation (p=0.011) but the global test was not significant (0.272), so we decided to keep it in the model and assumed approximate proportionality.
- The lifestyle model was adjusted for participants' age, body mass index, alcohol consumption, smoking, and folic acid supplementation.
- The fully adjusted model was adjusted for participants' age, migration background, cohabitation status, body mass index, alcohol consumption, smoking, and folic acid supplementation. Cohabitation status and folic acid supplementation showed a possible violation (p=0.013 and p=0.037) but the global test was not significant (0.218), so we decided to keep it in the model and assumed approximate proportionality.

Educational level of men:

- The confounder model was adjusted for participants' age.
- The demographic model was adjusted for participants' age, migration background, and cohabitation status. Cohabitation status showed a possible violation (p=0.028) but the global test was not significant (0.192), so we decided to keep it in the model and assumed approximate proportionality.
- The lifestyle model was adjusted for participants' age, body mass index, alcohol consumption, and smoking. Alcohol consumption was not significant.
- The fully adjusted model was adjusted for participants' age, migration background, cohabitation status, body mass index, and smoking. Cohabitation status showed a possible violation (p=0.045) but the global test was not significant (0.330), so we decided to keep it in the model and assumed approximate proportionality.

Educational level of women and men combined:

- The confounder model was adjusted for age of women and men, and parity. Parity and the global test showed possible violation (both p<0.001) so we decided to include parity as a stratification factor in the model. After stratification, the global test was not significant (p=0.94). Parity was then treated as a stratification factor. Age of men was not significant.
- The demographic model was adjusted for age of women, parity (stratified), migration background of women and men, and cohabitation status. Cohabitation status showed a possible violation (p=0.027) but the global test was not significant (0.298), so we decided to keep it in the model and assumed approximate proportionality. Migration background of men was not significant.
- The lifestyle model was adjusted for age of women, parity (stratified), body mass index of women and men, alcohol consumption of women and men, smoking of women and men, and folic acid supplementation. Body mass index of women (p=0.025), folic acid (p<0.001) and the global test (p<0.001) showed a possible violation. Folic acid was included as a stratification factor in the model. After stratification, the body mass index of women (p=0.0093) and alcohol consumption of men (p=0.438) showed a possible violation, but the global test was not significant (0.267) so we decided to keep them in the model and assumed approximate proportionality. Alcohol consumption of men was not significant.
- The fully adjusted model was adjusted for age of women, parity (stratified), migration background of women, cohabitation status, body mass index of women and men, alcohol consumption of women, smoking of women and men, and folic acid supplementation (stratified). Body mass index of women showed a possible violation (p=0.012), but the global test was not significant (p=0.247) so we decided to keep it in the model and assumed approximate proportionality.

Household income:

- The confounder model was adjusted for participant's age and parity. Parity and the global test showed possible violation (p=0.027 and p=0.012) so we decided to include parity as a stratification factor in the model. After stratification, the global test was not significant (p=0.24). Parity was then treated as a stratification factor.
- The demographic model was adjusted for participants' age, parity (stratified), migration background and cohabitation status. Cohabitation status showed a possible violation (p=0.039) but the global test was not significant (0.330), so we decided to keep it in the model and assumed approximate proportionality.
- The lifestyle model was adjusted for participants' age, parity (stratified), body mass index, alcohol consumption, smoking, and folic acid supplementation.
- The fully adjusted model was adjusted for participants' age, parity (stratified), migration background, cohabitation status, body mass index, alcohol consumption, smoking, and folic acid supplementation. Cohabitation status showed a possible violation (p=0.043) but the global test was not significant (0.341), so we decided to keep it in the model and assumed approximate proportionality.

© 2025 Boxem AJ et al. *JAMA Network Open*.

eTable 8. Associations of Poverty, Educational Level of Women and Men, and Household Income With Fecundability Ratios, Excluding Top 5% of Time to Pregnancy

	Total, n	Not conceived, n (%)	Basic model FR (95% CI)	Confounder model FR (95% CI)	Demographic factors model FR (95% CI)	Lifestyle factors model FR (95% CI)	Fully adjusted model FR (95% CI)
**Poverty**	2,280	174 (7.6)					
Yes	131	8 (6.1)	0.77 (0.64-0.92)	0.76 (0.64-0.92)	0.90 (0.74-1.09)	0.79 (0.66-0.96)	0.90 (0.74-1.10)
No	2,149	166 (7.7)	Reference	Reference	Reference	Reference	Reference
**Educational level of women**	2,395	194 (8.1)					
Low	118	13 (11.0)	0.78 (0.64-0.96)	0.73 (0.60-0.90)	0.82 (0.67-1.01)	0.78 (0.63-0.96)	0.86 (0.70-1.07)
Middle	560	45 (8.0)	0.84 (0.76-0.93)	0.77 (0.69-0.85)	0.83 (0.75-0.93)	0.81 (0.73-0.91)	0.87 (0.78-0.98)
High	1,717	136 (7.9)	Reference	Reference	Reference	Reference	Reference
**Educational level of men**	2,270	158 (7.0)					
Low	189	17 (9.0)	0.83 (0.71-0.97)	0.82 (0.70-0.96)	0.88 (0.75-1.04)	0.80 (0.68-0.94)	0.85 (0.72-1.00)
Middle	674	50 (7.4)	0.85 (0.77-0.93)	0.82 (0.75-0.91)	0.86 (0.78-0.95)	0.81 (0.73-0.89)	0.83 (0.75-0.92)
High	1,407	91 (6.5)	Reference	Reference	Reference	Reference	Reference
**Educational level of women and men**	2,270	158 (7.0)					
Both without high	493	36 (7.3)	0.81 (0.73-0.90)	0.73 (0.65-0.82)	0.79 (0.69-0.89)	0.76 (0.66-0.86)	0.77 (0.68-0.88)
Only women with high	370	31 (8.4)	0.87 (0.77-0.99)	0.86 (0.76-0.97)	0.83 (0.79-1.01)	0.85 (0.75-0.97)	0.86 (0.76-0.98)
Only men with high	122	5 (4.1)	0.92 (0.76-1.11)	0.89 (0.74-1.08)	0.93 (0.77-1.13)	0.92 (0.75-1.12)	0.93 (0.76-1.13)
Both with high	1,285	86 (6.7)	Reference	Reference	Reference	Reference	Reference
**Household income in euros per month**	2,352	190 (8.1)					
Less than 3,000	531	64 (12.1)	0.77 (0.67-0.89)	0.72 (0.62-0.84)	0.83 (0.70-0.97)	0.77 (0.66-0.90)	0.88 (0.74-1.04)
3,000-5,999	1,482	104 (7.0)	1.00 (0.88-1.13)	0.96 (0.85-1.09)	0.96 (0.85-1.09)	1.00 (0.88-1.14)	1.00 (0.88-1.13)
Equal or more than 6,000	339	22 (6.5)	Reference	Reference	Reference	Reference	Reference

Poverty:

- The confounder model was adjusted for participants' age and parity. Parity and the global test showed possible violation (p=0.029 and p=0.012) so we decided to include parity as a stratification factor in the model. After stratification, the global test was still significant (p=0.017), but visual inspection of the residuals showed that these were relatively constant. Parity was treated as a stratification factor.
- The demographic model was adjusted for participants' age, parity (stratified), migration background, and cohabitation status.
- The lifestyle model was adjusted for participants' age, parity (stratified), body mass index, alcohol consumption, smoking, and folic acid supplementation.
- The fully adjusted model was adjusted for participants' age, parity (stratified), migration background, cohabitation status, body mass index, alcohol consumption, smoking, and folic acid supplementation.

© 2025 Boxem AJ et al. *JAMA Network Open*.

Educational level of women:

- The confounder model was adjusted for participants' age and parity. Parity and the global test showed possible violation (p=0.05 and p=0.03) so we decided to include parity as a stratification factor in the model. After stratification, the global test was not significant (p=0.051). Parity was treated as a stratification factor.
- The demographic model was adjusted for participants' age, parity (stratified), migration background, and cohabitation status. Age and the global test showed possible violation (p=0.032 and p=0.046), but visual inspection of the residuals showed that these were relatively constant. We decided to keep age in the model and assumed approximate proportionality.
- The lifestyle model was adjusted for participants' age, parity (stratified), body mass index, alcohol consumption, smoking, and folic acid supplementation.
- The fully adjusted model was adjusted for participants' age, parity (stratified), migration background, cohabitation status, body mass index, alcohol consumption, smoking, and folic acid supplementation.

Educational level of men:

- The confounder model was adjusted for participants' age.
- The demographic model was adjusted for participants' age, migration background, and cohabitation status. Migration background was not significant.
- The lifestyle model was adjusted for participants' age, body mass index, alcohol consumption, and smoking. Alcohol consumption was not significant.
- The fully adjusted model was adjusted for participants' age, cohabitation status, body mass index, and smoking.

Educational level of women and men combined:

- The confounder model was adjusted for age of women and men, and parity. Parity and the global test showed possible violation (both p<0.001) so we decided to include parity as a stratification factor in the model. After stratification, the global test was not significant (p=0.30). Parity was treated as a stratification factor. Age of men was not significant.
- The demographic model was adjusted for age of women, parity (stratified), migration background of women and men, and cohabitation status. Cohabitation status showed a possible violation (p=0.036) but the global test was not significant (0.080), so we decided to keep it in the model and assumed approximate proportionality. Cohabitation status and migration background of men were not significant.
- The lifestyle model was adjusted for age of women, parity (stratified), body mass index of women and men, alcohol consumption of women and men, smoking of women and men, and folic acid supplementation. Folic acid supplementation and the global test showed possible violation (both p<0.001) so we decided to include folic acid supplementation as a stratification factor in the model. After stratification, the global test was not significant (p=0.1279). Folic acid was treated as a stratification factor. Body mass index and alcohol consumption of men were not significant.
- The fully adjusted model was adjusted for age of women, parity (stratified), migration background of women body mass index of women, alcohol consumption of women, smoking of women and men, and folic acid supplementation (stratified). Smoking of men showed a possible violation (p=0.028) but the global test was not significant (0.099), so we decided to keep it in the model and assumed approximate proportionality.

Household income:

- The confounder model was adjusted for participant's age and parity. Parity and the global test showed possible violation (p=0.029 and p=0.049) so we decided to include parity as a stratification factor in the model. After stratification, age was significant (0.042) but the global test was not significant (p=0.210) so we decided to keep age in the model. Parity was treated as a stratification factor.
- The demographic model was adjusted for participants' age, parity (stratified), migration background and cohabitation status. Age was significant (0.036) but the global test was not significant (0.420) so we decided to keep it in the model.
- The lifestyle model was adjusted for participants' age, parity (stratified), body mass index, alcohol consumption, smoking, and folic acid supplementation.
- The fully adjusted model was adjusted for participants' age, parity (stratified), migration background, cohabitation status, body mass index, alcohol consumption, smoking, and folic acid supplementation.

© 2025 Boxem AJ et al. *JAMA Network Open*.

eTable 9. Associations of Poverty, Educational Level of Women and Men, and Household Income With Fecundability Ratios, Including Only Dutch Participants

	Total, n	Not conceived, n (%)	Basic model FR (95% CI)	Confounder model FR (95% CI)	Demographic factors model FR (95% CI)	Lifestyle factors model FR (95% CI)	Fully adjusted model FR (95% CI)
**Poverty**	1,485	108 (7.3)					
Yes	45	2 (4.4)	0.67 (0.49-0.91)	0.66 (0.49-0.90)	0.73 (0.53-1.01)	0.67 (0.48-0.92)	0.71 (0.51-0.99)
No	1,440	106 (7.4)	Reference	Reference	Reference	Reference	Reference
**Educational level of women**	1,536	111 (7.2)					
Low	62	1 (1.6)	0.92 (0.71-1.19)	0.88 (0.67-1.15)	0.94 (0.71-1.24)	0.95 (0.72-1.25)	0.98 (0.73-1.32)
Middle	321	21 (6.5)	0.83 (0.73-0.94)	0.77 (0.67-0.88)	0.78 (0.68-0.90)	0.81 (0.70-0.94)	0.84 (0.72-0.98)
High	1,153	89 (7.7)	Reference	Reference	Reference	Reference	Reference
**Educational level of men**	1,426	84 (5.9)					
Low	86	5 (5.8)	0.87 (0.69-1.09)	0.83 (0.65-1.04)	0.85 (0.68-1.08)	0.81 (0.64-1.03)	0.82 (0.65-1.04)
Middle	369	24 (6.5)	0.82 (0.73-0.93)	0.79 (0.70-0.90)	0.81 (0.71-0.92)	0.77 (0.68-0.88)	0.77 (0.68-0.88)
High	971	55 (5.7)	Reference	Reference	Reference	Reference	Reference
**Educational level of women and men**	1,118	67 (6.0)					
Both without high	194	9 (4.6)	0.77 (0.65-0.91)	0.72 (0.61-0.86)	0.80 (0.68-0.95)	0.81 (0.67-0.98)	0.74 (0.62-0.88)
Only women with high	150	13 (8.7)	0.85 (0.70-1.02)	0.84 (0.70-1.02)	0.85 (0.71-1.02)	0.84 (0.69-1.02)	0.80 (0.66-0.96)
Only men with high	56	1 (1.8)	1.13 (0.86-1.49)	1.05 (0.80-1.39)	1.14 (0.86-1.50)	1.12 (0.84-1.50)	1.06 (0.80-1.41)
Both with high	718	44 (6.1)	Reference	Reference	Reference	Reference	Reference
**Household income in euros per month**	1,509	111 (7.4)					
Less than 3,000	245	25 (10.2)	0.74 (0.61-0.89)	0.67 (0.55-0.81)	0.71 (0.58-0.87)	0.72 (0.58-0.88)	0.69 (0.57-0.85)
3,000-5,999	1,022	73 (7.1)	0.96 (0.83-1.11)	0.90 (0.77-1.04)	0.90 (0.77-1.04)	0.92 (0.79-1.07)	0.91 (0.78-1.06)
Equal or more than 6,000	242	13 (5.4)	Reference	Reference	Reference	Reference	Reference

Poverty:

- The confounder model was adjusted for participants' age and parity. Parity and the global test showed possible violation (p=0.019 and p=0.046) so we decided to include parity as a stratification factor in the model. After stratification, the global test was not significant (p=0.36). Parity was treated as a stratification factor.
- The demographic model was adjusted for participants' age, parity (stratified), and cohabitation status. Cohabitation status and the global test showed possible violation (p=0.0053 and p=0.0163) so we decided to include it as a stratification factor in the model. After stratification, the global test was not significant (p=0.34). Cohabitation status was treated as a stratification factor.
- The lifestyle model was adjusted for participants' age, parity (stratified), body mass index, alcohol consumption, smoking, and folic acid supplementation. Folic acid supplementation showed possible violation (p=0.0069), but the global test was not significant (p=0.0685). However, visual inspection of the residuals showed that these were not constant so we decided to include folic acid supplementation as a stratification factor in the model. After stratification, the global test was not significant (p=0.68). Folic acid supplementation was treated as a stratification factor.
- The fully adjusted model was adjusted for participants' age, parity (stratified), cohabitation status (stratified), body mass index, alcohol consumption, smoking, and folic acid supplementation (stratified).

© 2025 Boxem AJ et al. *JAMA Network Open*.

Educational level of women:

- The confounder model was adjusted for participants' age and parity. Parity and the global test showed possible violation (p=0.015 and p=0.026) so we decided to include parity as a stratification factor in the model. After stratification, the global test was not significant (p=0.194). Parity was treated as a stratification factor.
- The demographic model was adjusted for participants' age, parity (stratified), migration background, and cohabitation status. Cohabitation status and the global test showed possible violation (p=0.0025 and p=0.0022) so we decided to include it as a stratification factor in the model. After stratification, the global test was not significant (p=0.075). Cohabitation status was treated as a stratification factor.
- The lifestyle model was adjusted for participants' age, parity (stratified), body mass index, alcohol consumption, smoking, and folic acid supplementation. Folic acid supplementation showed a possible violation (p=0.018) but the global test was not significant (0.112). However, visual inspection showed a deviation, so we decided to include folic acid supplementation as a stratification factor in the model. After stratification, the global test was not significant (0.56).
- The fully adjusted model was adjusted for participants' age, parity (stratified), cohabitation status (stratified), body mass index, alcohol consumption, smoking, and folic acid supplementation (stratified).

Educational level of men:

- The confounder model was adjusted for participants' age.
- The demographic model was adjusted for participants' age, and cohabitation status. Cohabitation status showed a possible violation (p=0.024) but the global test was not significant (0.180), and visual inspection showed no deviating pattern of the residuals so we decided to keep it in the model and assumed approximate proportionality.
- The lifestyle model was adjusted for participants' age, body mass index, alcohol consumption, and smoking. body mass index and alcohol consumption were not significant.
- The fully adjusted model was adjusted for participants' age, migration background, cohabitation status, and smoking. Cohabitation status showed a possible violation (p=0.025) but the global test was not significant (0.271), and visual inspection showed no deviating pattern of the residuals so we decided to keep it in the model and assumed approximate proportionality.

Educational level of women and men combined:

- The confounder model was adjusted for age of women and men, and parity. Parity and the global test showed possible violation (both p<0.001) so we decided to include parity as a stratification factor in the model. After stratification, the global test was not significant (p=0.87). Parity was treated as a stratification factor. Age of women and men was not significant.
- The demographic model was adjusted for parity (stratified), and cohabitation status. Cohabitation status was not significant.
- The lifestyle model was adjusted for age of women and men, parity (stratified), body mass index of women and men, alcohol consumption of women and men, smoking of women and men, and folic acid supplementation. Folic acid supplementation, alcohol consumption of women and the global test showed possible violation (p=0.0014, p=0.0408 and p=0.0164) so we decided to include it as a stratification factor in the model. After stratification, the global test was not significant (p=0.293), but alcohol consumption of women was significant (p=0.018). Visual showed no deviating pattern of the residuals so we decided to keep it in the model and assumed approximate proportionality. Folic acid supplementation was treated as a stratification factor. Body mass index of women and men, alcohol consumption of women and men, and smoking of women were not significant.
- The fully adjusted model was adjusted for parity (stratified), smoking of men, and folic acid supplementation (stratified).

Household income:

- The confounder model was adjusted for participant's age and parity. Parity and the global test showed possible violation (p=0.0068 and p=0.0013) so we decided to include parity as a stratification factor in the model. After stratification, the global test was not significant (p=0.069). Parity was treated as a stratification factor.
- The demographic model was adjusted for participants' age, parity (stratified), and cohabitation status. Cohabitation status was not significant.
- The lifestyle model was adjusted for participants' age, parity (stratified), body mass index, alcohol consumption, smoking, and folic acid supplementation. Smoking was not significant.
- The fully adjusted model was adjusted for participants' age, parity (stratified), body mass index, alcohol consumption, and folic acid supplementation.

© 2025 Boxem AJ et al. *JAMA Network Open*.

eTable 10. Associations of Poverty, Educational Level of Women and Men, and Household Income With Risks of Subfertility

	Total, n	Subfertile, n (%)	**Basic model** RR (95% CI)	**Demographic factors model** RR (95% CI)	**Lifestyle factors model** RR (95% CI)
**Poverty**	2,628	883 (33.6)			
Yes	169	85 (50.3)	1.55 (1.32-1.82)	1.16 (0.97-1.38)	1.28 (1.07-1.53)
No	2,459	798 (32.5)	Reference	Reference	Reference
**Educational level of women**	2,770	957 (42.2)			
Low	147	63 (42.9)	1.35 (1.10-1.64)	1.24 (1.01-1.51)	1.20 (0.97-1.48)
Middle	664	270 (40.7)	1.28 (1.14-1.43)	1.28 (1.14-1.44)	1.25 (1.10-1.41)
High	1,959	624 (31.9)	Reference	Reference	Reference
**Educational level of men**	2,599	838 (32.2)			
Low	232	90 (38.8)	1.33 (1.12-1.60)	1.24 (1.03-1.48)	1.34 (1.12-1.61)
Middle	785	288 (36.7)	1.26 (1.12-1.42)	1.26 (1.11-1.42)	1.31 (1.16-1.49)
High	1,582	460 (29.1)	Reference	Reference	Reference
**Educational level of women and men**	2,599	838 (32.2)			
Both without high	584	226 (38.7)	1.37 (1.20-1.56)	1.39 (1.20-1.61)	1.34 (1.15-1.56)
Only women with high	433	152 (35.1)	1.24 (1.06-1.44)	1.16 (1.00-1.35)	1.16 (0.99-1.35)
Only men with high	148	54 (36.5)	1.29 (1.03-1.62)	1.29 (1.03-1.61)	1.21 (0.96-1.53)
Both with high	1,434	406 (28.3)	Reference	Reference	Reference
**Household income in euros per month**	2,717	933 (34.3)			
Less than 3,000	658	301 (45.7)	1.51 (1.27-1.79)	1.26 (1.04-1.52)	1.34 (1.12-1.60)
3,000-5,999	1,677	516 (30.8)	1.01 (0.86-1.20)	1.05 (0.89-1.23)	1.01 (0.85-1.19)
Equal or more than 6,000	382	116 (30.4)	Reference	Reference	Reference

We tested the linearity assumption for age at enrollment in all models and found a significant quadratic term, which affected the estimates. Therefore, all models include a quadratic age term.

Poverty:

- The demographic model was adjusted for participants’ age, parity, migration background, and cohabitation status.
- The lifestyle model was adjusted for participants’ age, parity, body mass index, alcohol consumption, smoking, and folic acid supplementation.

Educational level of women:

- The demographic model was adjusted for participants’ age, migration background, and cohabitation status.
- The lifestyle model was adjusted for participants’ age, body mass index, alcohol consumption, smoking, and folic acid supplementation.

© 2025 Boxem AJ et al. *JAMA Network Open*.

Educational level of men:

- The demographic model was adjusted for participants' age, migration background, and cohabitation status. Migration background was not significant.
- The lifestyle model was adjusted for participants' age, body mass index, alcohol consumption, and smoking. Alcohol consumption was not significant.

Educational level of women and men combined:

- The demographic model was adjusted for age of women and men, migration background of women and men, and cohabitation status. Migration background of men was not significant.
- The lifestyle model was adjusted for age of women and men, body mass index of women and men, alcohol consumption of women and men, smoking of women and men, and folic acid supplementation. Body mass index of men and folic acid supplementation were not significant.

Household income:

- The demographic model was adjusted for participants' age, migration background and cohabitation status.
- The lifestyle model was adjusted for participants' age, body mass index, alcohol consumption, smoking, and folic acid supplementation.

© 2025 Boxem AJ et al. *JAMA Network Open*.

eTable 11. Associations of Poverty, Educational Level of Women and Men, and Household Income With Risks of Subfertility, Excluding Couples Undergoing Assisted Reproductive Technology

	Total, n	Subfertile, n (%)	Basic model RR (95% CI)	Confounder model RR (95% CI)	Demographic factors model RR (95% CI)	Lifestyle factors model RR (95% CI)	Fully adjusted model RR (95% CI)
**Poverty**	2,367	622 (26.3)					
Yes	158	74 (46.8)	1.89 (1.57-2.26)	1.57 (1.29-1.91)	1.22 (0.99-1.50)	1.35 (1.10-1.66)	1.10 (0.90-1.36)
No	2,209	548 (24.8)	Reference	Reference	Reference	Reference	Reference
**Educational level of women**	2,487	674 (27.1)					
Low	134	50 (37.3)	1.57 (1.24-1.99)	1.41 (1.10-1.81)	1.26 (0.99-1.61)	1.21 (0.93-1.57)	1.12 (0.88-1.44)
Middle	603	209 (34.7)	1.46 (1.27-1.68)	1.49 (1.29-1.72)	1.33 (1.14-1.54)	1.31 (1.12-1.53)	1.21 (1.03-1.41)
High	1,750	415 (23.7)	Reference	Reference	Reference	Reference	Reference
**Educational level of men**	2,356	595 (25.3)					
Low	204	62 (30.4)	1.43 (1.14-1.80)	1.43 (1.14-1.79)	1.27 (1.01-1.61)	1.50 (1.18-1.90)	1.32 (1.04-1.67)
Middle	727	230 (31.6)	1.49 (1.29-1.72)	1.54 (1.33-1.78)	1.43 (1.23-1.66)	1.57 (1.35-1.83)	1.46 (1.24-1.70)
High	1,425	303 (21.3)	Reference	Reference	Reference	Reference	Reference
**Educational level of women and men**	2,356	595 (25.3)					
Both without high	542	184 (33.9)	1.65 (1.41-1.94)	1.63 (1.37-1.95)	1.47 (1.22-1.78)	1.47 (1.21-1.79)	1.46 (1.21-1.77)
Only women with high	389	108 (27.8)	1.35 (1.11-1.64)	1.29 (1.07-1.56)	1.21 (1.00-1.46)	1.25 (1.02-1.52)	1.24 (1.02-1.50)
Only men with high	131	37 (28.2)	1.37 (1.02-1.84)	1.36 (1.02-1.82)	1.28 (0.96-1.71)	1.20 (0.89-1.62)	1.20 (0.89-1.61)
Both with high	1,294	266 (20.6)	Reference	Reference	Reference	Reference	Reference
**Household income in euros per month**	2,441	657 (26.9)					
Less than 3,000	592	235 (39.7)	1.77 (1.42-2.20)	1.66 (1.33-2.07)	1.33 (1.04-1.70)	1.43 (1.13-1.81)	1.16 (0.91-1.49)
3,000-5,999	1,506	345 (22.9)	1.02 (0.82-1.27)	1.05 (0.85-1.29)	1.03 (0.83-1.27)	0.98 (0.79-1.21)	0.97 (0.78-1.19)
Equal or more than 6,000	343	77 (22.4)	Reference	Reference	Reference	Reference	Reference

We tested the linearity assumption for age at enrollment in all models and found a significant quadratic term, which affected the estimates. Therefore, all models include a quadratic age term.

Poverty:

- The confounder model was adjusted for participants' age and parity. The demographic model was adjusted for participants' age, parity, migration background, and cohabitation status.
- The lifestyle model was adjusted for participants' age, parity, body mass index, alcohol consumption, smoking, and folic acid supplementation. Folic acid supplementation was not significant.
- The fully adjusted model was adjusted for participants' age, parity, migration background, cohabitation status, body mass index, alcohol consumption, and smoking.

Educational level of women:

- The confounder model was adjusted for participants' age and parity.
- The demographic model was adjusted for participants' age, parity, migration background, and cohabitation status.

© 2025 Boxem AJ et al. *JAMA Network Open*.

- The lifestyle model was adjusted for participants' age, parity, body mass index, alcohol consumption, smoking, and folic acid supplementation. Folic acid supplementation was not significant.
- The fully adjusted model was adjusted for participants' age, parity, migration background, cohabitation status, body mass index, alcohol consumption, and smoking.

Educational level of men:

- The confounder model was adjusted for participants' age.
- The demographic model was adjusted for participants' age, migration background, and cohabitation status.
- The lifestyle model was adjusted for participants' age, body mass index, alcohol consumption, and smoking. Alcohol consumption was not significant.
- The fully adjusted model was adjusted for participants' age, migration background, cohabitation status, body mass index, and smoking.

Educational level of women and men combined:

- The confounder model was adjusted for age of women and men, and parity.
- The demographic model was adjusted for age of women and men, parity, migration background of women and men, and cohabitation status. Migration background of men was not significant.
- The lifestyle model was adjusted for age of women and men, parity, body mass index of women and men, alcohol consumption of women and men, smoking of women and men, and folic acid supplementation. Body mass index of women and men and folic acid supplementation were not significant.
- The fully adjusted model was adjusted for age of women and men, parity, migration background of women, cohabitation status, alcohol consumption of women and men, and smoking of women and men.

Household income:

- The confounder model was adjusted for participant's age and parity.
- The demographic model was adjusted for participants' age, parity, migration background and cohabitation status.
- The lifestyle model was adjusted for participants' age, parity, body mass index, alcohol consumption, smoking, and folic acid supplementation. Folic acid supplementation was not significant.
- The fully adjusted model was adjusted for participants' age, parity, migration background, cohabitation status, body mass index, alcohol consumption, and smoking.

© 2025 Boxem AJ et al. *JAMA Network Open*.

eTable 12. Associations of Poverty, Educational Level of Women and Men, and Household Income With Risks of Subfertility, Excluding Top 5% of Time to Pregnancy

	Total, n	Subfertile, n (%)	Basic model	Confounder model	Demographic factors model	Lifestyle factors model	Fully adjusted model
			RR (95% CI)	RR (95% CI)	RR (95% CI)	RR (95% CI)	RR (95% CI)
**Poverty**	2,496	751 (30.1)					
Yes	145	61 (42.1)	1.43 (1.17-1.75)	1.29 (1.04-1.59)	1.13 (0.91-1.41)	1.25 (1.01-1.55)	1.11 (0.89-1.39)
No	2,351	690 (29.3)	Reference	Reference	Reference	Reference	Reference
**Educational level of women**	2,631	818 (31.1)					
Low	135	51 (37.8)	1.30 (1.04-1.63)	1.27 (0.99-1.61)	1.21 (0.95-1.53)	1.17 (0.92-1.50)	1.14 (0.89-1.44)
Middle	615	221 (35.9)	1.24 (1.09-1.41)	1.29 (1.13-1.47)	1.22 (1.06-1.39)	1.20 (1.04-1.38)	1.15 (1.00-1.32)
High	1,881	546 (29.0)	Reference	Reference	Reference	Reference	Reference
**Educational level of men**	2,469	708 (28.7)					
Low	218	76 (34.9)	1.32 (1.08-1.61)	1.31 (1.07-1.59)	1.24 (1.01-1.52)	1.35 (1.10-1.66)	1.32 (1.08-1.61)
Middle	725	228 (31.4)	1.19 (1.04-1.36)	1.24 (1.08-1.42)	1.19 (1.03-1.37)	1.25 (1.09-1.45)	1.24 (1.08-1.43)
High	1,526	404 (26.5)	Reference	Reference	Reference	Reference	Reference
**Educational level of women and men**	2,469	708 (28.7)					
Both without high	535	177 (33.1)	1.27 (1.09-1.48)	1.31 (1.10-1.55)	1.27 (1.07-1.50)	1.23 (1.03-1.47)	1.23 (1.03-1.47)
Only women with high	408	127 (31.1)	1.20 (1.01-1.42)	1.17 (0.99-1.39)	1.13 (0.95-1.34)	1.14 (0.95-1.35)	1.12 (0.94-1.34)
Only men with high	136	42 (30.9)	1.19 (0.91-1.55)	1.21 (0.92-1.57)	1.18 (0.90-1.54)	1.14 (0.87-1.50)	1.14 (0.87-1.50)
Both with high	1,390	362 (26.0)	Reference	Reference	Reference	Reference	Reference
**Household income in euros per month**	2,581	797 (30.9)					
Less than 3,000	593	236 (39.8)	1.46 (1.20-1.77)	1.41 (1.16-1.72)	1.24 (1.00-1.54)	1.31 (1.07-1.60)	1.14 (0.91-1.41)
3,000-5,999	1,622	461 (28.4)	1.04 (0.87-1.25)	1.07 (0.89-1.28)	1.06 (0.89-1.27)	1.02 (0.85-1.23)	1.02 (0.85-1.22)
Equal or more than 6,000	366	100 (27.3)	Reference	Reference	Reference	Reference	Reference

We tested the linearity assumption for age at enrollment in all models and found a significant quadratic term, which affected the estimates. Therefore, all models include a quadratic age term.

Poverty:

- The confounder model was adjusted for participants' age and parity. Parity was not significant.
- The demographic model was adjusted for participants' age, migration background, and cohabitation status.
- The lifestyle model was adjusted for participants' age, body mass index, alcohol consumption, smoking, and folic acid supplementation.
- The fully adjusted model was adjusted for participants' age, migration background, cohabitation status, body mass index, alcohol consumption, smoking, and folic acid supplementation.

Educational level of women:

- The confounder model was adjusted for participants' age and parity. Parity was not significant.

© 2025 Boxem AJ et al. *JAMA Network Open*.

- The demographic model was adjusted for participants' age, migration background, and cohabitation status.
- The lifestyle model was adjusted for participants' age, body mass index, alcohol consumption, smoking, and folic acid supplementation.
- The fully adjusted model was adjusted for participants' age, migration background, cohabitation status, body mass index, alcohol consumption, smoking, and folic acid supplementation.

Educational level of men:
- The confounder model was adjusted for participants' age.
- The demographic model was adjusted for participants' age, migration background, and cohabitation status. Migration background and cohabitation status were not significant.
- The lifestyle model was adjusted for participants' age, body mass index, alcohol consumption, and smoking. Alcohol consumption was not significant.
- The fully adjusted model was adjusted for participants' age, body mass index, and smoking.

Educational level of women and men combined:
- The confounder model was adjusted for age of women and men, and parity. Parity was not significant.
- The demographic model was adjusted for age of women and men, migration background of women and men, and cohabitation status. Migration background of men and cohabitation status were not significant.
- The lifestyle model was adjusted for age of women and men, body mass index of women and men, alcohol consumption of women and men, smoking of women and men, and folic acid supplementation. Body mass index of men was not significant.
- The fully adjusted model was adjusted for age of women and men, migration background of women, body mass index of women, alcohol consumption of women and men, smoking of women and men, and folic acid supplementation.

Household income:
- The confounder model was adjusted for participant's age and parity. Parity was not significant.
- The demographic model was adjusted for participants' age, migration background and cohabitation status.
- The lifestyle model was adjusted for participants' age, body mass index, alcohol consumption, smoking, and folic acid supplementation.
- The fully adjusted model was adjusted for participants' age, migration background, cohabitation status, body mass index, alcohol consumption, smoking, and folic acid supplementation.

© 2025 Boxem AJ et al. *JAMA Network Open*.

eTable 13. Associations of Poverty, Educational Level of Women and Men, and Household Income With Risks of Subfertility, Including Only Dutch Participants

	Total, n	Subfertile, n (%)	Basic model RR (95% CI)	Confounder model RR (95% CI)	Demographic factors model RR (95% CI)	Lifestyle factors model RR (95% CI)	Fully adjusted model RR (95% CI)
**Poverty**	1,632	490 (30.0)					
Yes	46	20 (43.5)	1.47 (1.05-2.06)	1.45 (1.03-2.05)	1.35 (0.95-1.91)	1.49 (1.06-2.10)	1.35 (0.96-1.92)
No	1,586	470 (29.6)	Reference	Reference	Reference	Reference	Reference
**Educational level of women**	1,689	514 (30.4)					
Low	69	21 (30.4)	1.07 (0.74-1.55)	1.07 (0.73-1.58)	1.04 (0.71-1.54)	1.01 (0.67-1.51)	0.94 (0.62-1.40)
Middle	354	133 (37.6)	1.32 (1.13-1.55)	1.41 (1.19-1.66)	1.40 (1.18-1.65)	1.27 (1.06-1.53)	1.25 (1.04-1.49)
High	1,266	360 (28.4)	Reference	Reference	Reference	Reference	Reference
**Educational level of men**	1,573	464 (29.5)					
Low	102	36 (35.3)	1.25 (0.94-1.65)	1.20 (0.90-1.59)	Not applicable	Not applicable	Not applicable
Middle	398	124 (31.2)	1.10 (0.92-1.31)	1.15 (0.97-1.36)	Not applicable	Not applicable	Not applicable
High	1,073	304 (28.3)	Reference	Reference	Reference	Reference	Reference
**Educational level of women and men**	1,232	352 (28.6)					
Both without high	212	73 (34.4)	1.30 (1.05-1.62)	1.35 (1.08-1.68)	1.35 (1.08-1.69)	1.20 (0.92-1.56)	1.27 (1.00-1.62)
Only women with high	165	49 (29.7)	1.13 (0.87-1.46)	1.10 (0.85-1.42)	1.10 (0.85-1.42)	1.08 (0.83-1.39)	1.08 (0.83-1.39)
Only men with high	67	22 (32.8)	1.24 (0.87-1.79)	1.30 (0.91-1.87)	1.30 (0.91-1.87)	1.20 (0.84-1.72)	1.20 (0.84-1.72)
Both with high	788	208 (26.4)	Reference	Reference	Reference	Reference	Reference
**Household income in euros per month**	1,659	504 (30.4)					
Less than 3,000	272	111 (40.8)	1.53 (1.19-1.95)	1.54 (1.21-1.98)	1.48 (1.13-1.93)	1.41 (1.10-1.82)	1.41 (1.10-1.82)
3,000-5,999	1,125	323 (28.7)	1.07 (0.86-1.34)	1.13 (0.91-1.40)	1.13 (0.91-1.40)	1.09 (0.87-1.35)	1.09 (0.87-1.35)
Equal or more than 6,000	262	70 (26.7)	Reference	Reference	Reference	Reference	Reference

We tested the linearity assumption for age at enrollment in all models and found a significant quadratic term, which affected the estimates. Therefore, all models include a quadratic age term.

Poverty:

- The confounder model was adjusted for participants' age and parity. Parity was not significant.
- The demographic model was adjusted for participants' age, and cohabitation status.
- The lifestyle model was adjusted for participants' age, body mass index, alcohol consumption, smoking, and folic acid supplementation.
- The fully adjusted model was adjusted for participants' age, cohabitation status, body mass index, alcohol consumption, smoking, and folic acid supplementation.

Educational level of women:

- The confounder model was adjusted for participants' age and parity. Parity was not significant.

© 2025 Boxem AJ et al. *JAMA Network Open*.

- The demographic model was adjusted for participants' age, and cohabitation status.
- The lifestyle model was adjusted for participants' age, body mass index, alcohol consumption, smoking, and folic acid supplementation.
- The fully adjusted model was adjusted for participants' age, cohabitation status, body mass index, alcohol consumption, smoking, and folic acid supplementation.

Educational level of men:

- The confounder model was adjusted for participants' age.

Educational level of women and men combined:

- The confounder model was adjusted for age of women and men, and parity. Age of women was not significant.
- The demographic model was adjusted for age of men, parity, and cohabitation status. Cohabitation status was not significant.
- The lifestyle model was adjusted for age of men, parity, body mass index of women and men, alcohol consumption of women and men, smoking of women and men, and folic acid supplementation. Body mass index of women and men and alcohol consumption of men were not significant.
- The fully adjusted model was adjusted for age of men, parity, alcohol consumption of women, smoking of women and men, and folic acid supplementation.

Household income:

- The confounder model was adjusted for participant's age and parity. Parity was not significant.
- The demographic model was adjusted for participants' age, and cohabitation status. Cohabitation status was not significant.
- The lifestyle model was adjusted for participants' age, body mass index, alcohol consumption, smoking, and folic acid supplementation.
- The fully adjusted model was adjusted for participants' age, body mass index, alcohol consumption, smoking, and folic acid supplementation.

© 2025 Boxem AJ et al. *JAMA Network Open*.

eTable 14. Associations of Poverty, Educational Level of Women and Men, and Household Income With Hazard Ratios of Miscarriage

	Total, n	Miscarriage, n (%)	Basic model HR (95% CI)	Confounder model HR (95% CI)	Demographic factors model HR (95% CI)	Lifestyle factors model HR (95% CI)	Fully adjusted model HR (95% CI)
**Poverty**	2,100	202 (9.6)					
Yes	127	7 (5.5)	0.55 (0.26-1.17)	0.58 (0.27-1.24)	Not applicable	Not applicable	Not applicable
No	1,973	195 (9.9)	Reference	Reference	Reference	Reference	Reference
**Educational level of women**	2,281	251 (11.0)					
Low	119	9 (7.6)	0.67 (0.34-1.30)	0.77 (0.39-1.51)	Not applicable	Not applicable	Not applicable
Middle	548	63 (11.5)	1.03 (0.78-1.38)	1.22 (0.91-1.64)	Not applicable	Not applicable	Not applicable
High	1,614	179 (11.1)	Reference	Reference	Reference	Reference	Reference
**Educational level of men**	2,148	226 (10.5)					
Low	192	19 (9.9)	0.93 (0.57-1.50)	0.95 (0.59-1.54)	Not applicable	Not applicable	Not applicable
Middle	628	66 (10.5)	0.98 (0.73-1.32)	1.02 (0.76-1.37)	Not applicable	Not applicable	Not applicable
High	1,328	141 (10.6)	Reference	Reference	Reference	Reference	Reference
**Educational level of women and men**	2,148	226 (10.5)					
Both without high	477	49 (10.3)	0.98 (0.70-1.36)	1.20 (0.85-1.71)	Not applicable	Not applicable	Not applicable
Only women with high	343	36 (10.5)	1.00 (0.69-1.45)	1.02 (0.70-1.48)	Not applicable	Not applicable	Not applicable
Only men with high	120	15 (12.5)	1.22 (0.71-2.09)	1.29 (0.75-2.21)	Not applicable	Not applicable	Not applicable
Both with high	1,208	126 (10.4)	Reference	Reference	Reference	Reference	Reference
**Household income in euros per month**	2,197	209 (9.5)					
Less than 3,000	525	47 (9.0)	1.03 (0.64-1.65)	1.15 (0.72-1.85)	Not applicable	Not applicable	Not applicable
3,000-5,999	1,351	134 (9.9)	1.14 (0.76-1.72)	1.27 (0.84-1.92)	Not applicable	Not applicable	Not applicable
Equal or more than 6,000	321	28 (8.7)	Reference	Reference	Reference	Reference	Reference

Poverty: The confounder model was adjusted for participants' age, parity, and history of miscarriage.
Educational level of women: The confounder model was adjusted for participants' age, parity, and history of miscarriage.
Educational level of men: The confounder model was adjusted for participants' age.
Educational level of women and men combined: The confounder model was adjusted for age of women and men, parity, and history of miscarriage.
Household income: The confounder model was adjusted for participant's age, parity, and history of miscarriage.

© 2025 Boxem AJ et al. *JAMA Network Open*.

eTable 15. Associations of Poverty, Educational Level of Women and Men, and Household Income With Hazard Ratios of Miscarriage, Excluding Couples Undergoing Assisted Reproductive Technology

	Total, n	Miscarriage, n (%)	Basic model HR (95% CI)	Confounder model HR (95% CI)	Demographic factors model HR (95% CI)	Lifestyle factors model HR (95% CI)	Fully adjusted model HR (95% CI)
**Poverty**	1,885	178 (9.4)					
Yes	118	6 (5.1)	0.51 (0.22-1.16)	0.53 (0.23-1.20)	Not applicable	Not applicable	Not applicable
No	1,767	172 (9.7)	Reference	Reference	Reference	Reference	Reference
**Educational level of women**	2,054	226 (11.0)					
Low	112	7 (6.2)	0.55 (0.26-1.19)	0.63 (0.29-1.35)	Not applicable	Not applicable	Not applicable
Middle	497	61 (12.3)	1.12 (0.84-1.51)	1.32 (0.97-1.79)	Not applicable	Not applicable	Not applicable
High	1,445	158 (10.9)	Reference	Reference	Reference	Reference	Reference
**Educational level of men**	1,953	209 (10.7)					
Low	173	18 (10.4)	0.96 (0.58-1.58)	0.99 (0.60-1.63)	Not applicable	Not applicable	Not applicable
Middle	583	62 (10.6)	0.97 (0.72-1.32)	1.02 (0.75-1.38)	Not applicable	Not applicable	Not applicable
High	1,197	129 (10.8)	Reference	Reference	Reference	Reference	Reference
**Educational level of women and men**	1,953	209 (10.7)					
Both without high	448	49 (10.9)	1.04 (0.74-1.46)	1.26 (0.89-1.80)	Not applicable	Not applicable	Not applicable
Only women with high	308	31 (10.1)	0.95 (0.64-1.42)	0.99 (0.66-1.47)	Not applicable	Not applicable	Not applicable
Only men with high	104	15 (14.4)	1.42 (0.83-2.45)	1.51 (0.88-2.59)	Not applicable	Not applicable	Not applicable
Both with high	1,093	114 (10.4)	Reference	Reference	Reference	Reference	Reference
**Household income in euros per month**	1,974	185 (9.4)					
Less than 3,000	473	38 (8.0)	0.93 (0.56-1.54)	1.05 (0.63-1.75)	Not applicable	Not applicable	Not applicable
3,000-5,999	1,211	122 (10.1)	1.18 (0.76-1.82)	1.31 (0.85-2.02)	Not applicable	Not applicable	Not applicable
Equal or more than 6,000	290	25 (8.6)	Reference	Reference	Reference	Reference	Reference

Poverty: The confounder model was adjusted for participants' age, parity, and history of miscarriage.
Educational level of women: The confounder model was adjusted for participants' age, parity, and history of miscarriage.
Educational level of men: The confounder model was adjusted for participants' age.
Educational level of women and men combined: The confounder model was adjusted for age of women and men, parity, and history of miscarriage.
Household income: The confounder model was adjusted for participant's age, parity, and history of miscarriage.

© 2025 Boxem AJ et al. *JAMA Network Open*.

eTable 16. Associations of Poverty, Educational Level of Women and Men, and Household Income With Hazard Ratios of Miscarriage, Including Only Dutch Participants

	Total, n	Miscarriage, n (%)	Basic model HR (95% CI)	Confounder model HR (95% CI)	Demographic factors model HR (95% CI)	Lifestyle factors model HR (95% CI)	Fully adjusted model HR (95% CI)
**Poverty**	1,355	136 (10.0)					
Yes	40	2 (5.0)	0.48 (0.12-1.95)	0.50 (0.12-2.05)	Not applicable	Not applicable	Not applicable
No	1,315	134 (10.2)	Reference	Reference	Reference	Reference	Reference
**Educational level of women**	1,422	155 (10.9)					
Low	61	4 (6.6)	0.59 (0.22-1.61)	0.62 (0.22-1.71)	Not applicable	Not applicable	Not applicable
Middle	290	36 (12.4)	1.17 (0.80-1.70)	1.27 (0.86-1.88)	Not applicable	Not applicable	Not applicable
High	1,071	115 (10.7)	Reference	Reference	Reference	Reference	Reference
**Educational level of men**	1,325	143 (10.8)					
Low	81	6 (7.4)	0.67 (0.29-1.55)	0.67 (0.29-1.54)	Not applicable	Not applicable	Not applicable
Middle	323	38 (11.8)	1.08 (0.74-1.58)	1.13 (0.77-1.65)	Not applicable	Not applicable	Not applicable
High	921	99 (10.7)	Reference	Reference	Reference	Reference	Reference
**Educational level of women and men**	1,042	114 (10.9)					
Both without high	175	17 (9.7)	0.90 (0.53-1.54)	1.03 (0.59-1.81)	Not applicable	Not applicable	Not applicable
Only women with high	129	18 (14.0)	1.32 (0.79-2.23)	1.38 (0.81-2.35)	Not applicable	Not applicable	Not applicable
Only men with high	55	7 (12.7)	1.22 (0.56-2.67)	1.27 (0.58-2.80)	Not applicable	Not applicable	Not applicable
Both with high	683	72 (10.5)	Reference	Reference	Reference	Reference	Reference
**Household income in euros per month**	1,384	139 (10.0)					
Less than 3,000	229	25 (10.9)	1.20 (0.67-2.15)	1.32 (0.73-2.37)	Not applicable	Not applicable	Not applicable
3,000-5,999	926	93 (10.0)	1.10 (0.68-1.77)	1.18 (0.73-1.91)	Not applicable	Not applicable	Not applicable
Equal or more than 6,000	229	21 (9.2)	Reference	Reference	Reference	Reference	Reference

Poverty: The confounder model was adjusted for participants' age, parity, and history of miscarriage.
Educational level of women: The confounder model was adjusted for participants' age, parity, and history of miscarriage.
Educational level of men: The confounder model was adjusted for participants' age.
Educational level of women and men combined: The confounder model was adjusted for age of women and men, parity, and history of miscarriage.
Household income: The confounder model was adjusted for participant's age, parity, and history of miscarriage.

© 2025 Boxem AJ et al. *JAMA Network Open*.

eTable 17. Associations of Poverty, Educational Level of Women and Men, and Household Income With Risks of Miscarriage

	Total, n	Miscarriage, n (%)	Basic model RR (95% CI)	Demographic factors model RR (95% CI)	Lifestyle factors model RR (95% CI)
**Poverty**	2,103	204 (9.7)			
Yes	127	7 (5.5)	0.55 (0.27-1.15)	Not applicable	Not applicable
No	1,976	197 (10.0)	Reference	Reference	Reference
**Educational level of women**	2,285	254 (11.1)			
Low	120	10 (8.3)	0.75 (0.41-1.38)	Not applicable	Not applicable
Middle	549	64 (11.7)	1.05 (0.80-1.37)	Not applicable	Not applicable
High	1,616	180 (11.1)	Reference	Reference	Reference
**Educational level of men**	2,151	228 (10.6)			
Low	192	19 (9.9)	0.93 (0.59-1.46)	Not applicable	Not applicable
Middle	629	67 (10.7)	1.00 (0.76-1.31)	Not applicable	Not applicable
High	1,330	142 (10.7)	Reference	Reference	Reference
**Educational level of women and men**	2,151	228 (10.6)			
Both without high	478	50 (10.5)	1.00 (0.74-1.37)	Not applicable	Not applicable
Only women with high	343	36 (10.5)	1.01 (0.71-1.43)	Not applicable	Not applicable
Only men with high	121	16 (13.2)	1.27 (0.78-2.06)	Not applicable	Not applicable
Both with high	1,209	126 (10.4)	Reference	Reference	Reference
**Household income in euros per month**	2,200	211 (9.6)			
Less than 3,000	527	49 (9.3)	1.07 (0.69-1.67)	Not applicable	Not applicable
3,000-5,999	1,351	134 (9.9)	1.14 (0.77-1.68)	Not applicable	Not applicable
Equal or more than 6,000	322	28 (8.7)	Reference	Reference	Reference

Poverty: The confounder model was adjusted for participants' age, parity, and history of miscarriage.
Educational level of women: The confounder model was adjusted for participants' age, parity, and history of miscarriage.
Educational level of men: The confounder model was adjusted for participants' age.
Educational level of women and men combined: The confounder model was adjusted for age of women and men, parity, and history of miscarriage.
Household income: The confounder model was adjusted for participant's age, parity, and history of miscarriage.

© 2025 Boxem AJ et al. *JAMA Network Open*.

eTable 18. Associations of Poverty, Educational Level of Women and Men, and Household Income With Risks of Miscarriage, Excluding Couples Undergoing Assisted Reproductive Technology

	Total, n	Miscarriage, n (%)	Basic model RR (95% CI)	Confounder model RR (95% CI)	Demographic factors model RR (95% CI)	Lifestyle factors model RR (95% CI)	Fully adjusted model RR (95% CI)
**Poverty**	1,888	180 (9.5)					
Yes	118	6 (5.1)	0.52 (0.23-1.14)	0.53 (0.24-1.15)	Not applicable	Not applicable	Not applicable
No	1,770	174 (9.8)	Reference	Reference	Reference	Reference	Reference
**Educational level of women**	2,058	229 (11.1)					
Low	113	8 (7.1)	0.64 (0.33-1.28)	0.73 (0.37-1.43)	Not applicable	Not applicable	Not applicable
Middle	498	62 (12.4)	1.13 (0.86-1.49)	1.32 (0.99-1.75)	Not applicable	Not applicable	Not applicable
High	1,447	159 (11.0)	Reference	Reference	Reference	Reference	Reference
**Educational level of men**	1,956	211 (10.8)					
Low	173	18 (10.4)	0.96 (0.60-1.53)	0.99 (0.62-1.57)	Not applicable	Not applicable	Not applicable
Middle	584	63 (10.8)	0.99 (0.75-1.32)	1.04 (0.78-1.38)	Not applicable	Not applicable	Not applicable
High	1,199	130 (10.8)	Reference	Reference	Reference	Reference	Reference
**Educational level of women and men**	1,956	211 (10.8)					
Both without high	449	50 (11.1)	1.07 (0.78-1.46)	1.28 (0.92-1.79)	Not applicable	Not applicable	Not applicable
Only women with high	308	31 (10.1)	0.97 (0.66-1.41)	0.99 (0.68-1.44)	Not applicable	Not applicable	Not applicable
Only men with high	105	16 (15.2)	1.46 (0.90-2.37)	1.55 (0.96-2.52)	Not applicable	Not applicable	Not applicable
Both with high	1,094	114 (10.4)	Reference	Reference	Reference	Reference	Reference
**Household income in euros per month**	1,977	187 (9.5)					
Less than 3,000	475	40 (8.4)	0.98 (0.61-1.58)	1.10 (0.69-1.76)	Not applicable	Not applicable	Not applicable
3,000-5,999	1,211	122 (10.1)	1.17 (0.78-1.77)	1.30 (0.86-1.95)	Not applicable	Not applicable	Not applicable
Equal or more than 6,000	291	25 (8.6)	Reference	Reference	Reference	Reference	Reference

Poverty: The confounder model was adjusted for participants' age, parity, and history of miscarriage.
Educational level of women: The confounder model was adjusted for participants' age, parity, and history of miscarriage.
Educational level of men: The confounder model was adjusted for participants' age.
Educational level of women and men combined: The confounder model was adjusted for age of women and men, parity, and history of miscarriage.
Household income: The confounder model was adjusted for participant's age, parity, and history of miscarriage.

© 2025 Boxem AJ et al. *JAMA Network Open*.

eTable 19. Associations of Poverty, Educational Level of Women and Men, and Household Income With Risks of Miscarriage, Including Only Dutch Participants

	Total, n	Miscarriage, n (%)	Basic model RR (95% CI)	Confounder model RR (95% CI)	Demographic factors model RR (95% CI)	Lifestyle factors model RR (95% CI)	Fully adjusted model RR (95% CI)
**Poverty**	1,355	136 (10.0)					
Yes	40	2 (5.0)	0.49 (0.13-1.91)	0.51 (0.13-1.97)	Not applicable	Not applicable	Not applicable
No	1,315	134 (10.2)	Reference	Reference	Reference	Reference	Reference
**Educational level of women**	1,422	155 (10.9)					
Low	61	4 (6.6)	0.61 (0.23-1.60)	0.65 (0.24-1.73)	Not applicable	Not applicable	Not applicable
Middle	290	36 (12.4)	1.16 (0.81-1.64)	1.26 (0.88-1.81)	Not applicable	Not applicable	Not applicable
High	1,071	115 (10.7)	Reference	Reference	Reference	Reference	Reference
**Educational level of men**	1,326	144 (10.9)					
Low	81	6 (7.4)	0.68 (0.31-1.51)	0.68 (0.31-1.50)	Not applicable	Not applicable	Not applicable
Middle	323	38 (11.8)	1.08 (0.76-1.54)	1.13 (0.80-1.60)	Not applicable	Not applicable	Not applicable
High	922	100 (10.8)	Reference	Reference	Reference	Reference	Reference
**Educational level of women and men**	1,042	114 (10.9)					
Both without high	175	17 (9.7)	0.92 (0.56-1.52)	1.04 (0.61-1.79)	Not applicable	Not applicable	Not applicable
Only women with high	129	18 (14.0)	1.32 (0.82-2.14)	1.38 (0.84-2.26)	Not applicable	Not applicable	Not applicable
Only men with high	55	7 (12.7)	1.21 (0.58-2.49)	1.27 (0.61-2.63)	Not applicable	Not applicable	Not applicable
Both with high	683	72 (10.5)	Reference	Reference	Reference	Reference	Reference
**Household income in euros per month**	1,384	139 (10.0)					
Less than 3,000	229	25 (10.9)	1.19 (0.69-2.06)	1.30 (0.75-2.26)	Not applicable	Not applicable	Not applicable
3,000-5,999	926	93 (10.0)	1.10 (0.70-1.72)	1.18 (0.75-1.84)	Not applicable	Not applicable	Not applicable
Equal or more than 6,000	229	21 (9.2)	Reference	Reference	Reference	Reference	Reference

Poverty: The confounder model was adjusted for participants' age, parity, and history of miscarriage.
Educational level of women: The confounder model was adjusted for participants' age, parity, and history of miscarriage.
Educational level of men: The confounder model was adjusted for participants' age.
Educational level of women and men combined: The confounder model was adjusted for age of women and men, parity, and history of miscarriage.
Household income: The confounder model was adjusted for participant's age, parity, and history of miscarriage.

© 2025 Boxem AJ et al. *JAMA Network Open*.
