# Supplementary material for: Poverty and Social Disadvantage in Women and Men and Fertility Outcomes
Source: JAMA Netw Open. 2025 Sep 19;8(9):e2532741. doi: 10.1001/jamanetworkopen.2025.32741 (PMC12449726; doi:10.1001/jamanetworkopen.2025.32741)
Supplement: Supplement 2. — Data Sharing Statement [file jamanetwopen-e2532741-s002.pdf]

Data Sharing Statement

Boxem. Poverty and Social Disadvantage in Women and Men and Fertility Outcomes. *JAMA Netw Open*. Published September 19, 2025. doi:10.1001/jamanetworkopen.2025.32741

Data
Data available: No

Additional Information
Explanation for why data not available: Data are available upon request to the corresponding author. Unrestricted data sharing is not allowed due to ethical consent and privacy restrictions.
